# Isotope-coded protein label based quantitative proteomic analysis reveals significant up-regulation of apolipoprotein A1 and ovotransferrin in the myopic chick vitreous

**DOI:** 10.1038/s41598-017-12650-7

**Published:** 2017-10-04

**Authors:** Feng-juan Yu, Thomas chuen Lam, Long-qian Liu, Rachel Ka-man Chun, Jimmy Ka-wai Cheung, King-kit Li, Chi-ho To

**Affiliations:** 1 Laboratory of Experimental Optometry, Centre for Myopia Research, School of Optometry, the Hong Kong Polytechnic University, Hung Hom, Kowloon Hong Kong; 2 Department of Ophthalmology, West China Hospital, Sichuan University, Chengdu, Sichuan China; 3 State Key Laboratory of Ophthalmology, Zhongshan Ophthalmic Center, Sun Yat-Sen University, Guangzhou, China

## Abstract

This study used isotope-coded protein label (ICPL) quantitative proteomics and bioinformatics analysis to examine changes in vitreous protein content and associated pathways during lens-induced eye growth. First, the vitreous protein profile of normal 7-day old chicks was characterized by nano-liquid chromatography electrospray ionization tandem mass spectrometry. A total of 341 unique proteins were identified. Next, myopia and hyperopia were induced in the same chick by attaching −10D lenses to the right eye and +10D lenses to the left eye, for 3 and 7 days. Protein expression in lens-induced ametropic eyes was analyzed using the ICPL approach coupled to LCMS. Four proteins (cystatin, apolipoprotein A1, ovotransferrin, and purpurin) were significantly up-regulated in the vitreous after 3 days of wearing −10D lenses relative to +10D lens contralateral eyes. The differences in protein expression were less pronounced after 7 days when the eyes approached full compensation. In a different group of chicks, western blot confirmed the up-regulation of apolipoprotein A1 and ovotransferrin in the myopic vitreous relative to both contralateral lens-free eyes and hyperopic eyes in separate animals wearing +10D lenses. Bioinformatics analysis suggested oxidative stress and lipid metabolism as pathways involved in compensated ocular elongation.

## Introduction

Myopia, the most common type of refractive error, has become a global public health issue^1,2^. High myopia frequently leads to severe pathological complications, such as cataract, retinal detachment, glaucoma, and other sight threatening conditions^3^. The increasing prevalence of myopia and its associated ocular complications is predicted to carry significant burden for individuals and society in the near future^4–6^. Although it is widely accepted that myopia is a multifactorial disease involving both genetic and environmental factors^7,8^, the exact mechanism underlying the aberrant eye growth remains unknown. Clarification of the specific mechanisms involved in the development of myopia is urgently needed to facilitate the development of effective prevention strategies or causal treatments for myopia.

Myopia has been extensively studied using environmentally-induced animal models, in various species, including monkey^9^, tree shrew^10^, chick^11^, and guinea pig^12^. They have provided good platforms to study GO (accelerating ocular growth and tune refractive status to myopia) and STOP signals (retarding ocular growth and tune refractive status to hyperopia) in regulating ocular growth and refractive error progression^13^. Of these models, the avian chick is the most established and commonly used species. It has the advantages of low breeding and maintenance costs, co-operative nature, and excellent optical components as well as fast and reproducible responses to induced manipulations^14,15^. In addition, availability of the complete genome of the chick (*Gallus gallus*) facilitates proteogenomics studies.

Vitreous humor is a transparent gel occupying the largest portion of the posterior eyeball cavity. An increased vitreous chamber depth (VCD) is the major contributing factor to the axial length elongation that underlies myopia^11^. It is believed that around 99% of vitreous volume is water with the remainder consisting of collagen fibers, hyalocytes, hyaluronic acid, lipids and low molecular weight substances or metabolites. The vitreous humor may function as a metabolic repository by storing proteins, amino acids, and metabolites that are biomolecules actively secreted to it or diffusing from surrounding tissues, such as retina, retinal pigmented epithelium, and the vasculature^16–19^. For instance, vitreal dopamine was suggested to originate from the retina. In chick eyes, vitreal dihydroxyphenylacetic acid (DOPAC), a dopamine metabolite, was found to diffuse freely in and out of the vitreous in chick eyes. The vitreal levels of DOPAC were reflecting and dependent on the retinal release of dopamine^20^. Normally, the vitreous fluid is protected by the blood-retinal barrier, and it has been suggested that changes in the protein composition of the vitreous occur in vitreoretinal and other ocular diseases^21–23^. Previous studies have reported the changes of vitreous protein composition in myopia by quantifying total protein concentration, and differentially expressed protein bands using gel approaches^24–26^. However, in part due to technical challenges, only limited progress has been made toward characterization of the vitreous proteome. Hence, comprehensive quantitation of low abundant vitreous proteins in ametropic chicks using a sensitive mass spectrometry (MS) approach may provide new insights to the mechanisms regulating myopic eye growth.

Advances in proteomic technology including labeling techniques, have dramatically improved large-scale identification and quantification of tissue proteomes in recent decades^27^. These techniques also have been applied to myopia models and several GO or STOP signals in different ocular tissues, mainly in the retina, have been identified during myopic growth^28–31^. Using conventional gel-based proteomic techniques, a novel apolipoprotein A1 was identified as a retinal STOP signal during eye growth. Its expression level decreased when the eye approached the completion of emmetropization during physiological eye development^32^, and in lens induced hyperopia in chick models^28^.

When compared with retina tissue, vitreous humor is known to contain fewer proteins which are expressed at lower concentration, thereby posing a technical challenge for conventional gel-based proteomic techniques. Isotope coded protein label (ICPL), a non-isobaric protein/peptide labeling technology coupled with liquid chromatography shotgun MS-based approach, has provided an economic, yet sensitive tool for quantifying proteins in highly complex mixtures^33,34^. This method applies different isotope tags to label lysine residues for up to four experimental groups, and measures relative abundances at the same time through MS peak intensity calculation, providing reproducible, accurate, quantitative results with high sequence coverage^35^. Using ICPL, this study characterizes the proteome of the chick vitreous and, via identification of differentially expressed proteins in response to +10/−10D lens wear.

**Figure 1:**
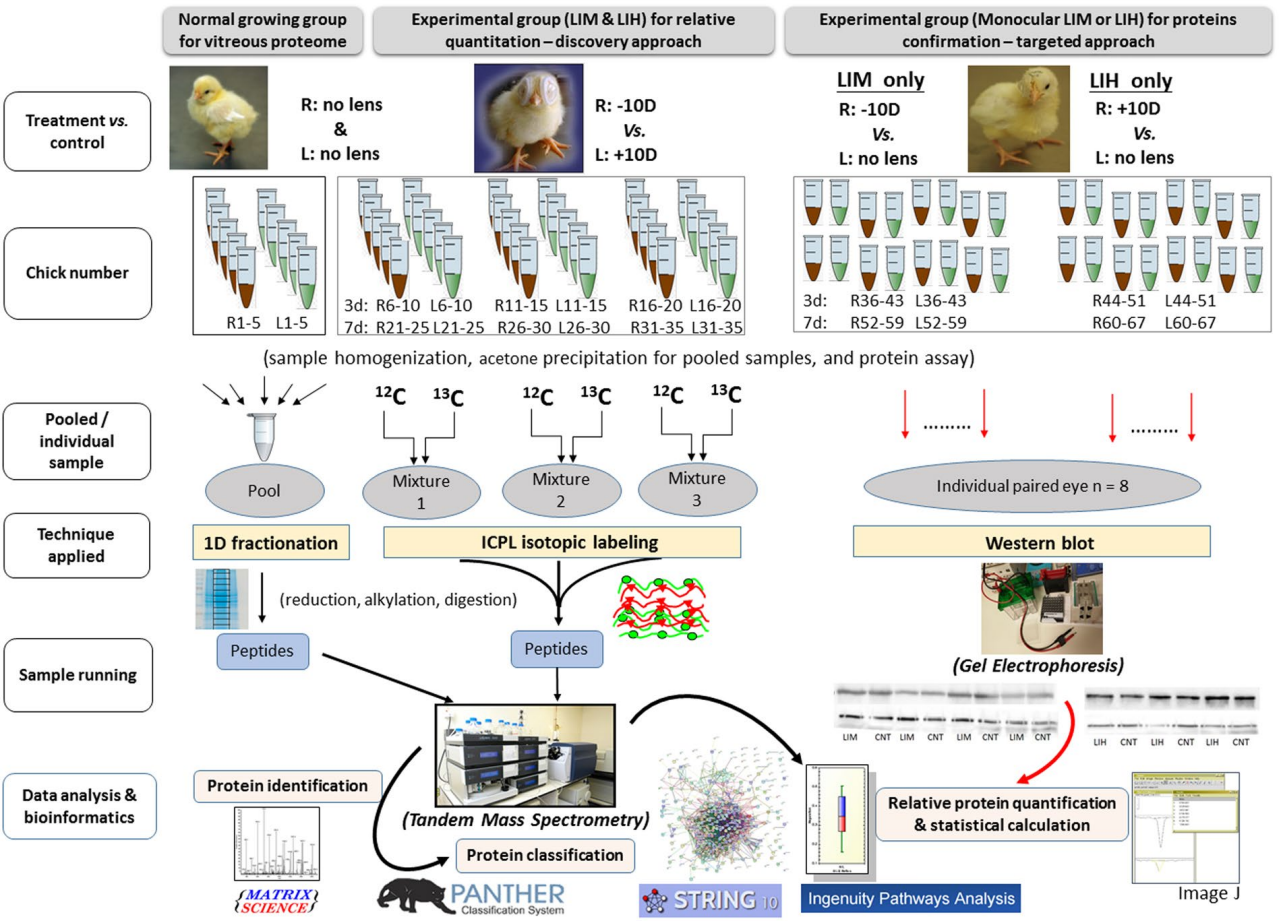
The experimental workflow and relevant technological platforms of three independent experiments for studying normal and compensated eye growth in chick vitreous proteome.

The vitreous proteome of normally growing chick eyes was first profiled using 1D gel fractionation coupled to nano-liquid chromatography electrospray ionization tandem Mass Spectrometry (nanoLC-ESI-MS/MS). After establishing a workable protocol adapted to vitreous fluid, additional samples were collected from bi-lateral lens wearing chicks (−10D and +10D lenses) for 3 and 7 days. Vitreous proteins from these treatment groups were extracted and labeled by ICPL technique for subsequent protein identification and relative quantification using the same nanoLC-ESI-MS/MS setup. Proteins of interest showing significant changes between −10D right eyes and +10D left eyes were selected and further validated by western blot in a separate group of chicks wearing monocular lenses (allowing an additional comparison with contralateral lens-free eyes). Using a novel bioinformatics tool, possible regulatory pathways of differentially expressed proteins involved in the compensated eye growth were suggested. An overview of our experimental set up is shown in Fig. 1.

## Results

### Biometric measurements in normal 7-day old chicks

Ocular component dimensions are presented in Table 1. In normal 7-day old chicks, no significant differences were found between right and left eye for any parameter (p > 0.05). Refractive errors remained slightly hyperopic (1.96 ± 0.82D). In accordance with this, the vitreous chamber depth (VCD) was 4.99 ± 0.10 mm, and axial length (AL) was 8.31 ± 0.11 mm. The vitreous protein concentration was 0.33 ± 0.10 μg/μl, with 100 µl lysis buffer added to each collected vitreous sample.

**Table 1:** Biometric values and refractive status of normally developed 7-day old chicks (n = 10).

Measurement	Mean ± SD (R)	Mean ± SD (L)	Average values
ACD (mm)	1.36 ± 0.04	1.35 ± 0.05	1.36 ± 0.04
Lens (mm)	1.96 ± 0.05	1.98 ± 0.03	1.97 ± 0.04
VCD (mm)	5.00 ± 0.11	4.97 ± 0.10	4.99 ± 0.10
Retina (mm)	0.24 ± 0.02	0.23 ± 0.01	0.24 ± 0.02
Choroid (mm)	0.13 ± 0.04	0.14 ± 0.04	0.13 ± 0.04
AL (mm)	8.32 ± 0.13	8.30 ± 0.09	8.31 ± 0.11
Refraction (D)	1.96 ± 0.76	1.96 ± 0.92	1.96 ± 0.89
Body weight (g)	**/**	**/**	76.55 ± 10.61

ACD: anterior chamber depth; VCD: vitreous chamber depth; AL: axial length (from the front of cornea to the back of vitreous chamber); R: right eyes; L: left eyes; Average values: average of right and left eye values.

### Protein identification and profiling of normal vitreous in 7-day old chicks

Using 1D SDS-PAGE fractionation coupled with LCMS, the total number of distinct proteins identified in the vitreous was 341 (MS results listed in appendix 1) based on the criteria that at least two peptides had to be identified for one positive protein identification using the chick IPI chick database (at a false discovery rate [FDR] <1.0%). The average sequence coverage of these identified proteins was 26.8 ± 15.4%. A protein network of all identified vitreous proteins was generated by using the STRING online database (http://string-db.org/), thereby creating an overall picture of protein information that also integrates direct and indirect interactions generated from known and predicted protein-protein associations. Closely related proteins are linked by colored lines indicating the type of interaction. Loose proteins are isolated proteins without known linkage to others. Large nodes indicate the availability of 3D protein structure, while small nodes indicate unknown 3D protein structure. (see Supplementary Fig. S1).

**Figure 2:**
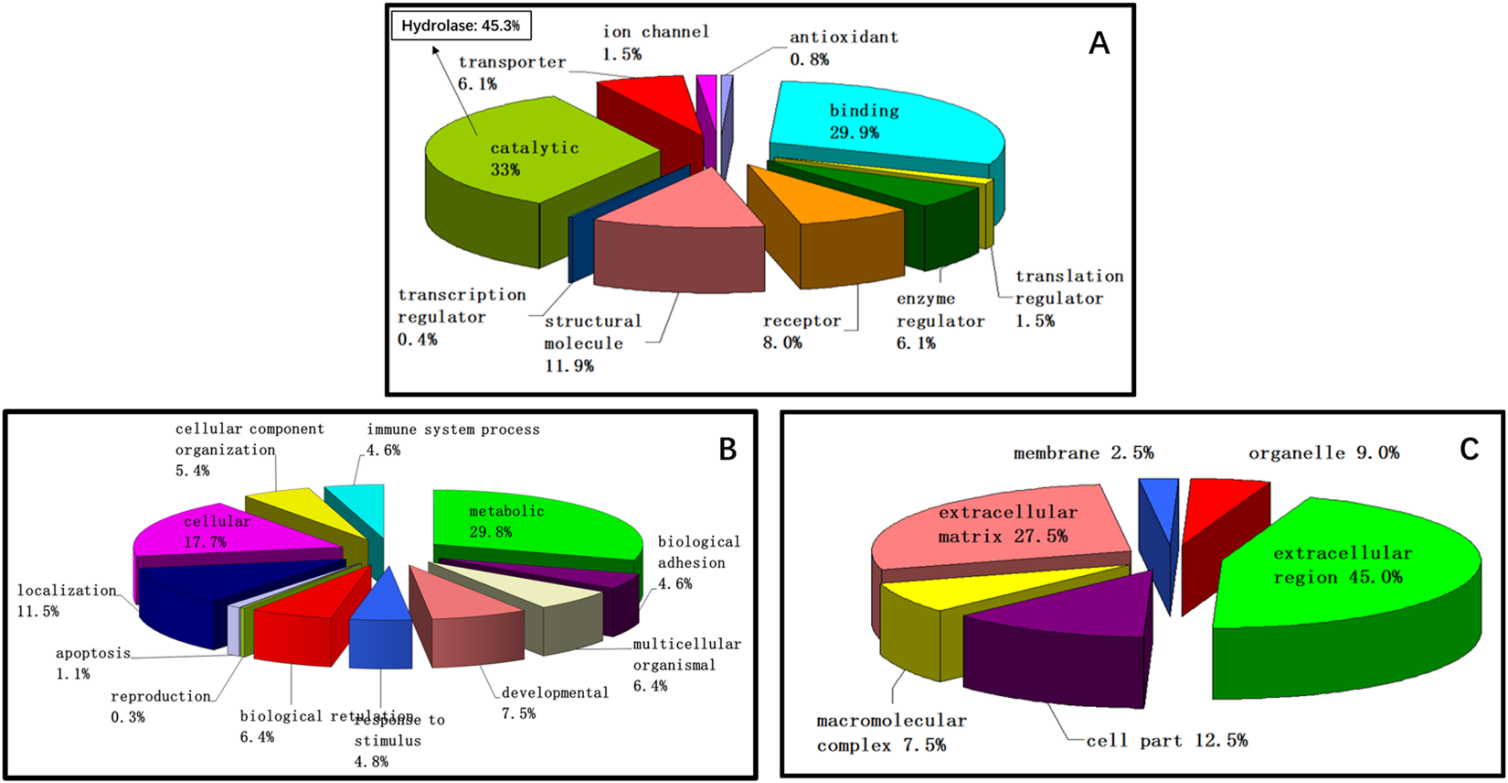
Gene ontology (GO) classifications of identified proteins in normal chick vitreous by (**A**) biological process (**B**) molecular functions (**C**) cellular components with the PANTHER^TM^ (Protein ANalysis THrough Evolutionary Relationships) Classification System.

All 341 proteins were then submitted for Gene ontology (GO) classification to the PANTHER^TM^ online system^36^. A total of 283 proteins (83% of all the identified proteins) were successfully mapped, and their properties are shown in Fig. 2. In terms of molecular function, “catalytic” (33.0%), and “binding” (29.9%) were the predominant groups followed by “structural molecule” (11.9%), “receptor” (8.0%), “transporter” (6.1%), and “enzyme regulator activities” (6.1%). Further sub-classification of “catalytic” activity revealed that “hydrolase” activity accounted for the largest subgroup (45.3%). Proteins can also be categorized according to their biological processes, and the identified proteins were classified to more than ten groups. “Metabolic process” (29.8%) and “cellular process” (17.7%) represented the largest proportions of this categorization. When proteins were classified according to their locations or cellular components, “extracellular region” (45.0%) and “extracellular matrix” (27.5%) accounted for more than 70% of all proteins. In addition, to better understand the hierarchical relations between over-represented or enriched functional classes of the vitreous proteome, lists of ‘protein class’ and ‘Reactome pathways’ (p < 0.05, Bonferroni correction for multiple testing) generated from PANTHER Overrepresentation Test (release 20170413) are shown as Supplementary Tables S2 and S3 respectively.

### Biometric measurements in lens-induced ametropic chicks

**Figure 3:**
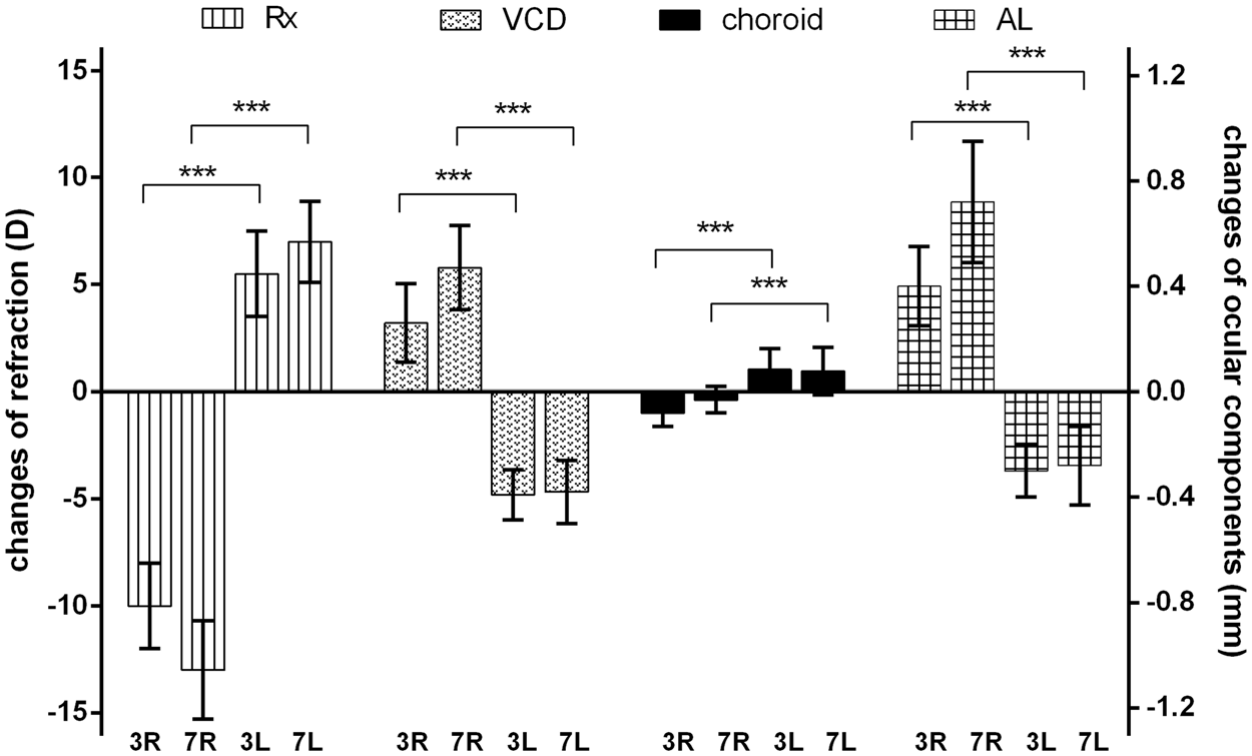
Changes in refraction (Rx, the left Y-axis) and ocular component measures (vitreous chamber depth, VCD; choroidal thickness and axial length, AL, the right Y-axis) after lens wear for 3 and 7 days. The values are differences between d0 and d3 or d7. Right (R) eyes wore −10D lenses while left (L) eye wore +10D lenses. The refraction and ocular components were significantly changed between the right and left eyes after lens wear for both treatment time points (paired t-test, **p < 0.01, ***p < 0.001, n = 15).

Changes in refraction and ocular components between days 0, days 3 and 7 of lens wear are shown in Fig. 3. Before lens application (d0), no significant interocular differences were detected for ocular components and refractive error (paired t-test, p > 0.05). After lens wear, significant refractive errors were induced. The negative lens induced more myopia after treatment for 7 days (d7-d0: −13.09 ± 2.07D) than 3 days (d3-d0: −10.03 ± 1.71D). Correspondingly, the axial lengths (AL) continued to elongate with time (d7-d0: 0.73 ± 0.24 mm; d3-d0: 0.39 ± 0.11 mm). VCD was also increased, but the absolute increase was smaller than for AL. Conversely, positive lenses gradually induced more hyperopia after treatment for 7 days (d7-d0: 7.15 ± 1.20D) than 3 days (d3-d0: 5.93 ± 1.83D). Similarly, axial lengths were equivalently decreased (d7-d0: −0.26 ± 0.17 mm; d3-d0: −0.29 ± 0.10 mm), and VCDs were decreased correspondingly. Opposite to the directions of AL and VCD changes, choroidal thickness decreased in response to the −10D, and increased in response to the +10D treatment. The magnitude of changes in choroid thickness was small, but significantly different between myopic right eye and hyperopic left eye at both time points.

### Protein quantitation in lens-induced ametropic chicks

After ICPL labeling and sample pooling, a total of 358 ± 23, and 339 ± 20 vitreous proteins (mean ± standard deviation, s. d.) were identified, after lens treatment for 3 and 7 days, respectively. The difference in number of identified proteins was neither significant (p > 0.05, independent t-test) between time points, nor between +10D and −10D lenses. Using the established protocol for MS identification and filters for relative quantification listed in the methods section (fold change > 1.47 and p < 0.05, t-test), four proteins, including ovotransferrin, apolipoprotein A1, cystatin, and purpurin were identified as differentially expressed (MS results listed in appendix 2). The basic MS information and fold changes of these four proteins are shown in Table 2. The four proteins were all up-regulated in myopic vitreous when compared to hyperopic vitreous after 3-day lens wear. However, after longer lens wear of 7-day, only levels of apolipoprotein A1 and purpurin remained statistically significant (p < 0.001), and only the expression level of purpurin met the pre-set criterion of a change > 1.49-fold. Although apolipoprotein A1 on day 7 was significant up-regulated in the t-test, the fold change (1.44-fold) was marginally below the pre-set cut-off value. The averaged fold changes of both ovotransferrin and cystatin also showed numerical up-regulated expressions on day 7 in the myopic vitreous, however, this difference neither reached significance in the t-test nor met the pre-set cut-off value.

**Table 2:** Changes of vitreous proteins with differential expression in lens induced ametropic chicks (myopia/hyperopia, n = 3). General information including molecular function, cellular component, and biological process are listed.

Protein name	Accession number	MW (kDa)	pI	Treatment for 3 days (−10D/ +10D)	Treatment for 7 days (−10D/ +10D)	Molecular Function	Cellular Component	Biological Process
**Ovotransferrin**	IPI00971372	79.6	7.8	**1.52 ± 0.12** ^***^	**1.23 ± 0.19**	Ferric iron binding	Extracellular region	Cellular iron ion homeostasis; iron ion transport
**Apolipoprotein A1**	IPI00580765	30.7	5.5	**1.59 ± 0.28** ^*^	**1.44 ± 0.13** ^***^	Beta-amyloid/cholesterol/phospholipid/HDL particle binding; Lipid/cholesterol/transmembrane transporter activity	Extracellular region (HDL secreted)	Blood circulation; lipid transport; lipcholesterol/steroid/sterol metabolism
**Cystatin**	IPI00576782	15.6	9.0	**1.65 ± 0.16** ^***^	**1.38 ± 0.14**	Protein binding; cysteine-type endopeptidase/protease/thiol protease inhibitor activity	Extracellular region	proteolysis
**Purpurin**	IPI00600469	22.2	4.6	**1.55 ± 0.10** ^***^	**1.49 ± 0.14** ^***^	Retinal/retinol binding; transporter activity	Extracellular/interphotoreceptor matrix	vitamin transport

***p < 0.001; and *p < 0.05

Ratios in bold denote proteins with significant up-regulation.

### Bioinformatics analysis by Ingenuity Pathway Analysis (IPA)

**Figure 4:**
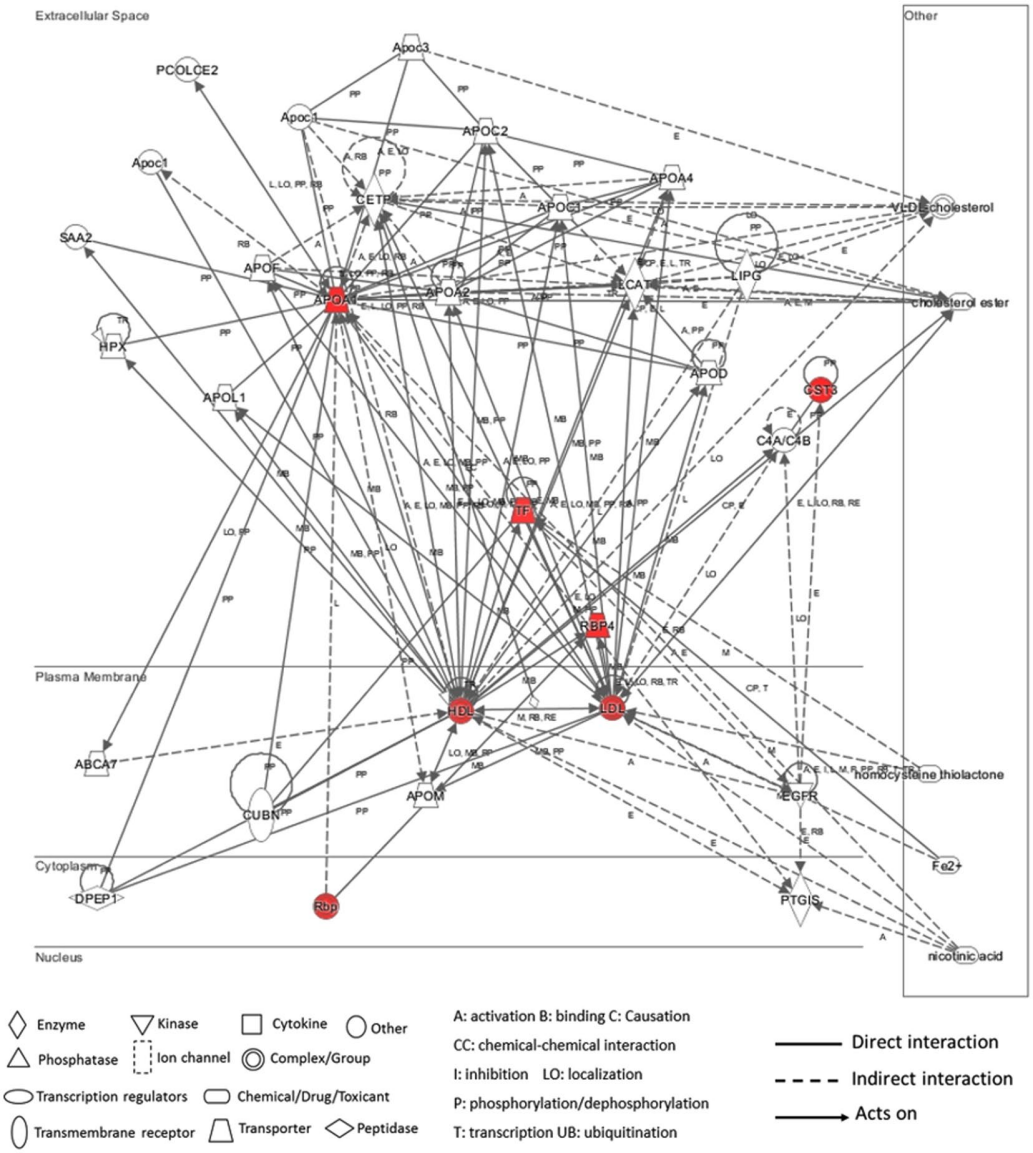
The network generated by IPA of the identified proteins altered during myopic ocular growth. Proteins in the network are represented by their gene symbols [TF: ovotransferrin; APOA1: apolipoprotein A1; CST3: cystatin; RBP4/RBP: retinol binding protein (purpurin-like protein); HDL: high density lipoprotein; and LDL: low density lipoprotein]. Four up-regulated proteins identified by LCMS and their close interactions with lipid metabolism (HDL and LDL) are highlighted in red. The white nodes are those identified from the IPA Knowledge Base that may be associated to the identified proteins through specific interactions.

Cloud-based IPA software was used to identify biological pathways that may be associated with these significantly up-regulated proteins. All four proteins were mapped with their corresponding gene IDs and their appropriate cellular component classification (i.e., extracellular space). The IPA-generated network shows the main known regulators of these mapped proteins and their interactions (Fig. 4). Apolipoprotein A1 and ovotransferrin were revealed as the central focus proteins with different interactions to many other molecules. Although no direct interactions were found among these four proteins, the IPA analysis revealed that they may interact indirectly via lipid metabolism pathways (specifically, via high and low density lipoproteins, HDL and LDL). In this IPA network analysis, nicotinic acid was indicated to be a key regulator of the indirect interactions between ovotransferrin, HDL, and LDL. Furthermore, cholesterol metabolism was revealed to be a potential common pathway involving all four differentially expressed proteins in chick vitreous.

### Western blot confirmation of the expression changes

**Figure 5:**
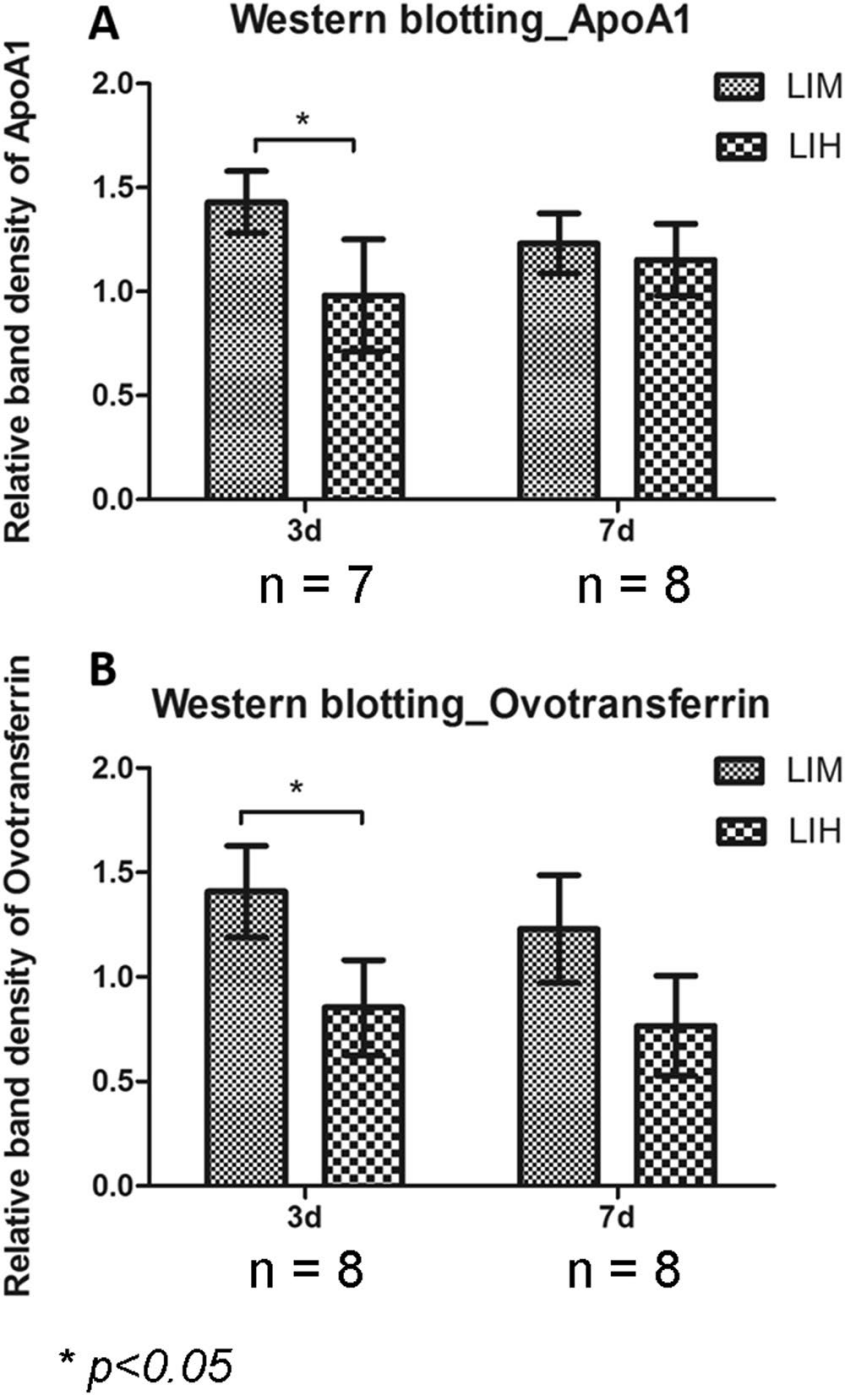
Western blot detections of protein changes in ametropic chick vitreous (mean ± standard deviation). The ratios in LIM and LIH were first normalized by their fellow control eyes (expressions in monocular myopia or hyperopia over control eyes). Expression changes of ApoA1 and ovotransferrin were significantly increased after −10D lenses inducement for 3 days, compared to +10D lenses inducement (*p < 0.05, paired t-test). No significant differences were detected after treatment for 7 days (p > 0.05).

After the discovery based proteomics, apolipoprotein A1 and ovotransferrin were found to be two most important nodes involved in regulation of eye growth. Owing to their biological significance and also to the availability of antibodies, these two proteins were chosen for further testing using western blot. For this follow up analysis, separate cohorts of chicks were treated with lenses over their right eyes and no lenses over their left eyes; −10D lenses for LIM, and +10D for LIH (Fig. 1). In LIM, the interocular differences of refraction were −8.21 ± 1.35D and −11.12 ± 2.23D after treatment for 3, and 7 days, respectively. The corresponding interocular differences in axial length were 0.31 ± 0.14 mm (3 days) and 0.64 ± 0.20 mm (7 days). In LIH, the interocular differences in refractive error were 5.85 ± 1.16D (3 days) and 7.13 ± 1.32D (7 days). The axial length in hyperopic side became relatively shorter compared to the control eye (3 days: −0.26 ± 0.10 mm; 7 days: −0.25 ± 0.11 mm). Figure 5 shows the relative expression level of apolipoprotein A1 (38 kDa) and ovotransferrin (80 kDa) in the ametropic chick vitreous in LIM and LIH and normalized to the contralateral, untreated control eyes (represented as monocular myopia/control or hyperopia/control). For western blot confirmation, apolipoprotein A1 (1.43 ± 0.15) and ovotransferrin (1.41 ± 0.22) were significantly up-regulated in myopic eyes relative to contralateral lens-free eyes (p < 0.05, n = 7 for apolipoprotein and n = 8 for ovotransferrin) after 3-day LIM treatment. However, the difference in hyperopic eye relative to contralateral lens-free eyes was not statistically significant. These changes also were not statistically significant anymore after 7 days in both time points (p > 0.05, n = 8). In addition, relative expression levels of apolipoprotein A1 and ovotransferrin were calculated as ratios between LIM and LIH, to allow for better comparison with the MS data. Similar to the MS, apolipoprotein A1 (1.39 ± 0.24-fold) and ovotransferrin (1.63 ± 0.42-fold) were both significantly up-regulated in myopic relative to hyperopic chick vitreous after 3 days of treatment (independent t test, p < 0.05, n = 7 for apolipoprotein and n = 8 for ovotransferrin). Numerical up-regulation of these two proteins was also observed in the LIM eyes after 7-day lens wear, but the changes were not statistically significant.

## Discussion

This study identified a reference dataset of the chick vitreous proteome using tandem shotgun MS for the first time. The results demonstrated that the vitreous might be a more complex tissue than commonly assumed. Although the vitreous is generally believed to be composed of ~99% water, our data suggested that the vitreous also contains diverse proteins for carrying out functional activities in the eyes that may have been overlooked in the past. Protein classifications by the PANTHER^TM^ and String databases also suggest that the chick vitreous proteome is involved in complex biological functions and processes. In accordance with the function of the vitreous, the majority of vitreous proteins are extracellular or extracellular matrix proteins, their roles including water retention, structural connection, support, and protection. Protein concentration in chick vitreous also indicated that vitreous protein composition may not be very simple in previous or the current study^25^. An even higher number of identified proteins than in this study was reported for the human vitreous^23,37^. The difference likely reflected the more sensitive multidimensional LCMS approach, which were used for the human studies. Both the human studies and this study reflect the vitreous as a biologically active tissue.

In chicks wearing −10D/+10D lenses, the eyes gradually compensated for the lens by adapting their growth. The overall changes of refraction and axial length were quite similar to previous reports on chicks wearing −10/ +10D lenses after birth^38,39^. Chick eyes with −10D lenses showed a faster elongation of VCD and AL, which was caused by the dual effects of inherent ocular growth and imposed negative lenses. In chick eyes with +10D lenses, the inherent ocular growth was antagonized by the +10D lens. As a result, the magnitude of change in VCD and AL after 7 days was less than after 3 days. Although the changes of choroidal thickness were significant between the right and left eyes at both time points, there was no significant difference in terms of magnitude after a longer wearing time (d3-d0 versus d7-d0). This is not surprising, as adaption of choroid thickness to imposed defocus occurs rapidly within 1 hour^40,41^. In addition, the response of choroidal thickness to lens induced defocus could wear off after lens wear for 2 days^40^.

In this study, vitreous total protein concentration after lens treatment did not change significantly, in agreement with another study^25^. However, four proteins (apolipoprotein A1, purpurin, cystatin, and ovotransferrin) in the vitreous with increased expression in LIM were identified as possible biomarkers for differential ocular growth. All four myopia-induced proteins are extracellular proteins and belong to the class of ‘binding proteins’. Three of them (ovotransferrin, apolipoprotein A1, and purpurin) have transporter functions, while cystatin is involved in proteolysis. Apolipoprotein A1 is the main component of HDL in plasma. Ovotransferrin belongs to the transferrin family, which is responsible for transferring ferric ions in many species. Purpurin belongs to the lipocalin family and has 50% sequence homology with human serum retinol-binding proteins (RBPs). Cystatin belongs to the family of endogenous protease inhibitors that inhibit endogenous and exogenous cysteine proteases from uncontrolled proteolysis^42^.

The proteomics results were validated by similar protein expression changes in western blot confirming apolipoprotein A1 and ovotransferrin were significantly up-regulated in myopic eyes after 3-day LIM treatment. Similar to the proteomic findings, these changes were not statistically significant anymore after 7 days suggesting that the expression changes occur early in myopia development. Different from the bi-lateral lens wear setup applied in relative proteomics quantification, −10D and +10D lenses were imposed on the right eyes while the left eyes were served as untouched control in this validation test. Hence, the difference in expression levels was mainly driven by up-regulation of these proteins in myopic eyes, with no statistically significant reduction of expression seen in the hyperopic eyes. These results further supported the significance of both apolipoprotein A1 and ovotransferrin as biomarkers for myopia.

**Apolipoprotein A1** is known as the major component in HDL, which is responsible for transporting cholesterol produced by extrahepatic cells to the liver for further processing or excretion from the body. Apart from this conventional role in lipid metabolism, a role for apolipoprotein A1 in ocular growth was revealed in recent studies. retinal apolipoprotein A1 was suggested to function as a STOP signal in eye growth as retinal and scleral apolipoprotein A1 levels were increased in chick LIH, potentially preventing the activation of transforming growth factor (TGF)-beta^28^. Consistent with those findings, scleral apolipoprotein A1 was down-regulated in LIM and up-regulated during recovery from LIM in tree shrews^43^. Similar to the finding in LIM, our previous study also demonstrated a steady decrease of retinal apolipoprotein A1 when the eyes become enlarged during normal development eye growth in chicks from postnatal day 3 to 20^32^ in chicks. In clinical studies, vitreous apolipoprotein A1 was reported to be up-regulated in diabetic patients^44,45^, which had been suggested that apolipoprotein A1 may guard the retina from oxidative stress in diabetic patients due to its role as a potent reactive oxygen species scavenger^46,47^. More recently, apolipoprotein A1 in chick choroid and sclera were found to have a novel regulatory feedback mechanism with retinoic acid, a well-established signal for regulating eye growth in myopia, in a concentration-dependent manner to control postnatal ocular growth^48^. Hence, the differential expression of vitreal apolipoprotein A1 expression that was seen with both discovery-based quantitative proteomic analysis and western blot in our study further confirm the involvement of this protein in excessive ocular growth. Although, the expression of apolipoprotein A1 in the myopic vitreous was found opposite to its expression in the retina, it may reflect a sequestration of a STOP signal from retina to vitreous. While in a previous study we showed that retinal dopamine is the source of vitreous dopamine and dopamine levels in retina and vitreous are regulated in concordant fashion, this may not be necessarily case for apolipoprotein A1 or other proteins. Importantly, proteins may reach the vitreous from not only the retina but also other surrounding regions. They may not only move via diffusion but also by more regulated transport mechanisms such as active pumping mechanism^49^. Further studies are needed to test these hypotheses and to elucidate the interplay between apolipoprotein A1 levels, retinoic acid levels, and TGF beta over time and anatomical location.

**Ovotransferrin** belongs to the transferrin family, which is responsible for transferring ferric ions in the tissues. It was reported to have anti-oxidative properties^50^ and was further found to act as a superoxide dismutase (SOD) mimic protein with SOD-like biological function, scavenging superoxide anions^51^. Therefore, the up-regulated ovotransferrin in the myopic vitreous may indicate increased oxidative stress during ocular elongation. Ovotransferrin function as an antioxidant was found to be affected by cholesterol^52^. Therefore, up-regulated ovotransferrin in ocular growth may also reflect lipid metabolism associated with ocular growth. In addition, both choroidal ovotransferrin gene and protein was suggested as a potential regulator of myopic eye growth^30,53^ and ovotransferrin mRNA expression was increased in the retina/RPE/choroid of chicks recovering from form-deprived myopia (FDM)^53^. The mechanism may be through slowing the rate of VCD elongation and inhibiting proteoglycan synthesis in the sclera^30^. In the current study, the change in vitreous ovotransferrin expression was opposite to that of choroid tissue during myopia development, similar to the findings of apolipoprotein A1 in vitreous and retina, again suggesting that the vitreous and other posterior parts of the eye may sequester retinal proteins when their functions are not required in the retina.

**Purpurin** belongs to the lipocalin family and has 50% sequence homology with human serum retinol-binding proteins (RBPs). It can bind and solubilize retinol and protect it from oxidation. Purpurin prolonged the survival of dissociated chick neural retina cells and ciliary ganglion cells, indicating its potential for trophic support in the nervous system^54,55^. In addition, purpurin was shown to participate in cell differentiation during early development of the zebrafish retina. An inhibition of transcriptional and translational expression of purpurin significantly reduced the eyeball size in zebrafish^56^. Gene expression in chick amacrine cell layer indicated purpurin was up-regulated following +7D lens wear for 24 hours^57^. However, in this study vitreous purpurin was found relatively up-regulated in LIM compared to LIH, potentially indicating antioxidant and trophic support roles during vitreous elongation and ocular growth, as well as possible sequestration in the vitreous. Moreover, its potential role in regulating retinoic acid metabolism during myopic eye growth should be further explored.

**Cystatin** belongs to the family of endogenous protease inhibitors that inhibit endogenous and exogenous cysteine proteases from uncontrolled proteolysis^42^. Its structure and function in chick is close to human cystatin C^58^. During normal retinal development in mammals, an increase in retinal cystatin C has been observed that may facilitate tissue growth and keep in check the degradation of photoreceptor outer segment proteins^59^. Cystatin C has also been identified as a novel TGF-beta receptor antagonist in a novel cystatin C-mediated feedback loop to inhibit TGF-beta signaling^60^. Moreover, Cystatin was found significantly up-regulated in chick retina/RPE/choroid during recovery from FDM at the gene level^53^. The up-regulated cystatin in vitreous during lens-induced ocular growth in the present study is in line with the observed increased retinal levels in physiological postnatal eye growth and suggests a role of vitreous cystatin in ocular growth, potentially related to the TGF-beta pathway.

Additionally, the IPA pathway analysis revealed cholesterol metabolism as closely related to the vitreous proteins implicated in compensated eye growth. IPA also revealed nicotinic acid, one of the water-soluble B vitamins, as a compound potentially modulating these proteins. Nicotinic acid is known to lower the concentration of lipoproteins, including LDL and lipoprotein(a) and raise protective factors, such as HDL in patients with hyperlipidemia^61^. This regulatory effect of nicotinic acid in lipoprotein metabolism may also play a role in treating myopia^62^. In addition, apolipoprotein A1 and cystatin may participate in myopia via TGF-beta signaling which is believed to control the ocular enlargement in myopic chicks^63–65^ and tree shrews^66^. Furthermore, previous studies have reported associations between TGF-beta and lipoproteins. LDL receptor-related protein-associated protein 1 (LRPPAP1) was characterized in knockout mice with increased TGF-beta levels. Additionally, an increased TGF-beta level was also shown with LRPAP1 mutations^67^. Using a novel transcriptomics approach in studying myopic chick retina/RPE/choroid, Riddell et al. recently detected candidate genes showing bi-directional expression changes across LIM and LIH groups and linked to pathways related to lipid metabolism^68^. Hence, elucidating the specific interactions of lipid metabolism with TGF-beta in myopia warrants further investigation.

The protein changes indicated an increase of oxidative stress in the myopic vitreous. The eye is an organ rich in reactive oxygen species with a high requirement of antioxidants to protect fatty acids. Oxidative stress has also been implied in other ocular diseases, including diabetic retinopathy^69^, age-related macular degeneration^70^, dry eye syndrome^71^, and cataract^72^. The retina has high oxygen consumption under physiological conditions, and a role for oxidative stress has been postulated in myopic ocular growth and accelerated axial elongation. In line with this hypothesis, increased levels of oxidized glutathione and related lipid peroxidation products were found in the vitreous of myopic patients compared with the controls^73^. Quantitative proteomics has also suggested dysregulation of mitochondrial oxidation in the retina of myopic mice^74^. The potential role of oxidative stress and its related contribution to the onset of lipid peroxidation in the myopic retina were reported^68,75^. As the metabolic activities in retina is vigorous and the vitreous is adjacent to the retina, the changes in detected protein candidates in vitreous may be due to the diffusion of retinal metabolic products as a response to induced defocus signals. This diffusion or interchange of proteins between the retina and vitreous would be possible if the blood-retinal barrier was disrupted^76^.

Interestingly, the number and magnitudes of protein expression changes involved in ocular growth were quite small. All protein regulations in this study were less than 2-fold even when there was a considerable ocular refraction difference in the same chick with 7-day lens treatment. Therefore, no linear correlation between the magnitude of protein expressions and the degree of ametropia could be identified. The four proteins we identified in the vitreous were also found in other ocular tissues involving growing process in chick or other models, suggesting a significant role in the development of myopia. However, the direction of expression changes may not be concordant in the vitreous and other ocular tissues, potentially indicating a sequestration function of the vitreous. Since temporal protein expression and protein-protein interactions are believed to be very dynamic in biological tissues, future research should focus on the proteomic changes in multiple layers from vitreous to sclera in the same eye at the same time point, to comprehensively study protein expression from the anterior to posterior eye.

The ametropic chick model in this study was using bi-directional −10D and +10D lenses on the same chick for identifying differentially expressed proteins because the number and the magnitude of protein expression changes in response to external defocus signals were relatively small from previous studies^29,31,77^. Furthermore, in a prior study, protein expression in experimental induced myopia or hyperopia was rarely changing in the same direction^28^. Hence, this study was designed to enlarge the interocular difference in protein expression and increase the sensitivity of the proteomic analysis. Using this approach, only significantly changed protein targets with opposite direction of expression or those with highly significant regulations in one side specific to LIM or LIH can be identified. Certainly, this design may exclude proteins showing the same direction in expression to both LIM and LIH. Since a large number of genes change expression in response to lens wear were found independent of positive or negative lenses refraction (similar trends were detected in both LIM and LIH)^57,68^, the design used in this study enriches growth direction-specific, differentially expressed proteins, at the expense of growth non direction-specific proteins. With this unique design, we therefore offered a more refined list of protein candidates for future follow up studies.

A potential limitation of the current study design is that contralateral eye effects (i.e. expression changes induced by one condition spreading to the other eye) may obscure expression changes. This experimental paradigm of using same animal contralateral eyes as control is commonly found in similar myopia studies. A gel-based proteomics study has also suggested that false positive results could be minimized when the comparison was made using two eyes of the same animal, whereas the same genetic makeup can be secured, instead of using different animals of the same ages^78^. Given that the contralateral eye effects are still possible, our design presumably reduces the true number of positive findings as we compared the relative protein fold changes between the two eyes. An additional limitation is that treatments for left and right eyes were homogeneous within cohorts, which theoretically may cause left/right biases^79^. Although this lateral asymmetry may not necessarily be reflected in vitreous protein expression levels, it can be addressed by assigning lens treatment in random fashion to left and right eyes within the same cohort. It is also worth noting that relatively strict pre-set thresholds were applied in this study for detecting significantly regulated proteins. The thresholds were rationally selected based on the variation and reproducibility of protein expression levels found in our control experiment, in which 95% confident interval can be secured using ICPL-based LCMS. These filters may have reduced the number of differential expressed proteins to be discovered. However, less strict thresholds will increase the risk for finding false positive candidates. Lastly, the sample pooling strategy was adopted to screen for differentially expressed proteins in our study mainly due to the low protein content in the vitreous. The benefits and limitations of this design were discussed in other proteomic studies^80,81^. To overcome the technical challenges associated with studying the vitreous proteome from individual biological samples, a label-free proteomics approach using more sensitive triple-TOF mass spectrometry may offer a solution in future studies^82^.

## Conclusion

In conclusion, this study is the first to analyze the chick vitreous proteome under normal conditions and in induced eye growth. Quantitative proteomic analysis identified four up-regulated proteins in LIM. Western blot validated apolipoprotein A1 and ovotransferrin as potential myopia markers in ocular development. Pathway analysis suggested changes in lipid metabolism together with the TGF-beta signal pathway may contribute to the progression of myopia. Increased oxidative stress in the vitreous during LIM was also suggested. Interestingly, expression changes in retina and vitreous move in opposite directions for multiple proteins during LIM. A potential explanation for these seemingly conflicting findings may be that the vitreous can act as a buffer for non-utilized STOP signals during LIM. By analyzing the chick vitreous proteome, this study provides new insights in the mechanisms leading to myopia.

## Methods

### Chick model

White Leghorn chicks (*Gallus gallus*), hatched from specific pathogen free eggs (SPF, Jinan, China), were housed in brooders under 12/12 hours light/dark cycle at approximately 25 °C. Food and water were provided *ad libitum*. All research with animals was conducted in compliance with the ARVO statement on the Use of Animals in Research. The experimental protocols used in this paper were approved by the Animal Subjects Ethics Sub-committee (ASESC) of the Hong Kong Polytechnic University. No gender preference was set in any of the experimental groupings. Refractive status and ocular components (anterior chamber depth, lens thickness, vitreous chamber depth, retinal thickness, choroidal thickness, and scleral thickness) were measured using a streak retinoscope and a high frequency A-scan ultrasound system with a 30 MHz transducer (Panametrics, Inc., Waltham, MA), respectively, before and after lens wear. The spherical equivalent (S.E.) was used to represent the refractive status (S.E. = spherical power +1/2 cylindrical power). Axial length was calculated from the front of cornea to the back of vitreous chamber.

For normal vitreous protein profiling, five chicks (10 eyes) aged 7-days without lens wear were pooled for the analysis. For comparing protein changes after lens-inducement, a new batch of chicks aged 4 to 5 days (d0) with similar body weight were mounted with −10D lenses (right eye) and +10D (left eye), respectively for 3 days and 7 days. Equal amounts of proteins from five samples of each group were pooled together to form representative lysates for myopic and hyperopic samples. Lenses were made of polymethylmethacrylate (PMMA) with an optical zone diameter of 11 mm and base curve of 6.7 mm. They were mounted using ring-shaped Velcro systems. Lenses were checked and cleaned daily to ensure secure attachment and clear vision.

### Vitreous protein extraction

Chicks were sacrificed with carbon dioxide overdose at the desired time points. The eyeball was enucleated and hemisected equatorially. The vitreous body including both the liquid and gel portions in the posterior pole was immediately extracted and frozen without contamination from other ocular tissues. Each vitreous was homogenized in a liquid nitrogen cooled Teflon freezer mill (Mikro-Dismembrator Braun Biotech, Melsungen, Germany) with 100 µl lysis buffer containing 7 M urea, 2 M thiourea, 30 mM Tris, 0.2% (w/v) Biolytes, 1% (w/v) dithiothreitol, 2% (w/v) CHAPS and 1% (w/v) ASB14 in protease inhibitor cocktail (Roche Applied Science, Basel, Switzerland). After recovering the soluble vitreous lysate, the sample was centrifuged at 16.1 × 1,000 g for 30 minutes at 4 °C. The supernatant was collected and protein concentration of each sample was measured using a 2-D Quant Kit (GE Healthcare Life Science, Marlborough, MA).

### Sample preparation for relative quantitation after +10/−10D treatment

A total of 15 chicks were used for relative quantitation of the lens treatment at each time point of 3d or 7d (Fig. 1). After individual homogenization of vitreous samples of LIM and LIH chicks, these 15 chicks were randomly assigned into groups of 5 to form 3 biological replicates (n = 3). Based on the protein assay, 20 µg vitreous protein from each right (LIM) and left eye (LIH) of five chicks were first pooled to form representative LIM and LIH lysates, respectively. After overnight acetone precipitation, protein pellets were re-dissolved in Guanidine-HCl (6 M, pH = 8.5). According to the protein assay, 50 µg of protein from the LIM and LIH samples was subjected to ICPL labeling according to the manufacturer’s instruction. Briefly, they were reduced with 0.2 M Tris (2-carboxyethayl) phosphine, alkylated with 0.4 mM iodoacetamide, and then labeled with ^12^C-Nic-reagent (ICPL_light, 105.0215 Da) and ^13^C-Nic-reagent (ICPL_heavy, 111.0419 Da) for the right (LIM) and left (LIH) eyes respectively. After incubation at room temperature for 2 hours, the labeling reaction was stopped by hydroxylamine. Samples from these two groups were then combined and further precipitated overnight in 80% ice-cooled acetone. The supernatant was removed and a protein pellet was obtained. The pellet was finally re-dissolved in 1 M Urea with 25 mM N H_4_HCO_3_ for in-solution digestion. Trypsin with a final ratio of 1:50 and endo-GluC with a final ratio 1:30 (total enzyme to protein, by weight) were used. Finally, collected peptides were re-dissolved in 0.1% formic acid for MS.

### Nano-liquid chromatography electrospray ionization tandem mass spectrometry

An Ultimate 3000 nanoHPLC system (Dionex, Sunnyvale, CA) was coupled with an ion trap mass spectrometer (HCTultra PTM discovery system, Bruker Daltonik GmbH, Bremen, Germany), which was operated in positive ion mode via a nanospray source. The peptides were first concentrated on a C18 PepMap trapping column (internal diameter of 300 μm × 5 mm, LC packing, Dionex, Sunnyvale, CA) and then separated on a C18 PepMap column (internal diameter of 75 μm × 150 mm, LC packing, Dionex, Sunnyvale, CA) with gradient conditions from 3–40% ACN in 0.08% formic acid at a flow rate of 200 nl/min. Precursor selection was set as 350–1500 m/z. The top four abundant ions were isolated for fragmentation by collision induced dissociation (CID). Generated MS spectra were searched against the chick International Protein Index database for protein identification via the Mascot search engine. WarpLC 1.2 Software (Bruker Daltonik GmbH, Bremen, Germany) was used to quantify protein expression levels, which were automatically calculated from the average peptide ratios (Light vs. Heavy) by comparing the relative intensities of the extracted ion chromatograms (EIC). Search parameters were as follows: 1) taxonomy was set to Chick; 2) carbamidomethylation of cysteine was the fixed modification, while methionine oxidation was a variable modification. In quantitative searching, ICPL_light (K), ICPL_light protein N-term, ICPL_heavy (K) and ICPL_heavy protein N-term were also specified as exclusive modifications; 3) trypsin and endo-GluC were selected as enzymes with a maximum of one allowed miscleaage. For protein identification in normal chick vitreous, K (lysine), R (arginine), D (aspartic acid) and E (glutamic acid) were set as the cleavage sites, while for protein identification in ametropic chick vitreous, R, D and E were set as K was labeled by ICPL isobaric tags and protected from trypsin cleavage; and 4) mass tolerance for peptide tolerance and MS/MS tolerance were set to 1.2 and 0.5 Da. Charge states of 2+ and 3+ were selected.

### Statistical analysis for differential expression

All biometric measurements and comparisons between the right and left eyes were analyzed by paired t-test. Measurements between different chicks were compared by independent t-test. P < 0.05 was considered as statistically significant in all cases. All values were presented as mean ± standard deviation. For protein identification, only proteins with at least two top-ranking peptides being significantly matched to a particular protein were considered as a positive identification. With regards to protein quantitation, two additional criteria were applied to detect a significantly changed protein target: 1) the average fold-change should be higher than 1.47 or smaller than 0.68. Based on our internal system variability tests for optimization, these cut-off values were selected based on the 95% confidence interval of the fold change of duplicate samples using pairwise values from all identified proteins at 1:1 lysate mixture using ICPL approach. 2) The difference of fold change should be significant (paired t-test, p < 0.05) between the right and left eyes.

### Data Availability

All data generated or analyzed during this study are included in this published article (and its Supplementary files). Raw MS dataset during the current study are available from the corresponding author on reasonable request.

## Electronic supplementary material

supplementary information

supplementary dataset protein identification
