# Supplementary material for: Isotope-coded protein label based quantitative proteomic analysis reveals significant up-regulation of apolipoprotein A1 and ovotransferrin in the myopic chick vitreous
Source: Sci Rep. 2017 Oct 4;7:12649. doi: 10.1038/s41598-017-12650-7 (PMC5627271; doi:10.1038/s41598-017-12650-7)

Isotope-coded protein label based quantitative proteomic analysis reveals significant up-regulation of apolipoprotein A1 and ovotransferrin in the myopic chick vitreous

*Feng-juan Yu[1], *Thomas chuen Lam[1], *Long-qian Liu[2], Rachel Ka-man Chun[1], Jimmy Ka-wai Cheung[1], King-kit Li[1], Chi-ho To[1, 3]*

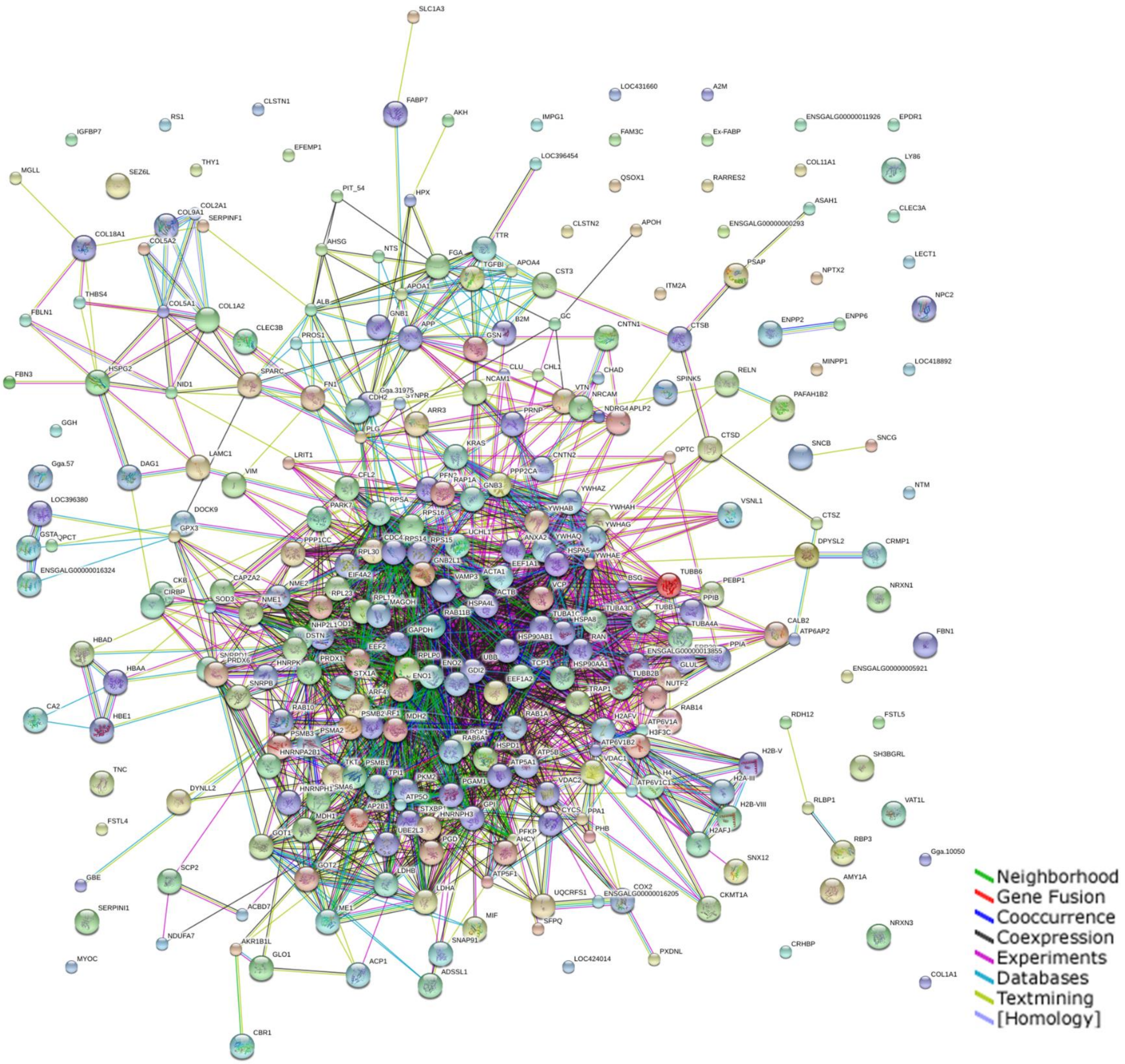

Supplementary Figure S1

	Gallus gallus (REF)	Client Text Box Input (▽ Hierarchy NEW! ?)				
PANTHER Protein Class	#	#	expected	Fold Enrichment	+/-	P value
Hsp90 family chaperone	4	3	.07	40.68	+	1.25E-02
↳chaperone	129	10	2.38	4.20	+	3.41E-02
cysteine protease inhibitor	7	4	.13	30.99	+	2.04E-03
amino acid kinase	6	3	.11	27.12	+	4.10E-02
anion channel	25	6	.46	13.02	+	1.71E-03
transfer/carrier protein	243	15	4.48	3.35	+	1.16E-02
Unclassified	7974	116	147.03	.79	-	0.00E00
transcription factor	934	3	17.22	< 0.2	-	4.65E-03

Supplementary Table S2

	Gallus gallus (REF)	Client Text Box Input (▽ Hierarchy NEW! ?)				
Reactome pathways	#	#	expected	Fold Enrichment	+/-	P value
Gluconeogenesis	17	10	.31	31.90	+	2.31E-09
↳Carbohydrate metabolism	25	10	.46	21.69	+	9.62E-08
↳Metabolism	50	12	.92	13.02	+	3.91E-07
Gluconeogenesis	20	11	.37	29.83	+	3.62E-10
↳Glucose metabolism	52	12	.96	12.52	+	6.06E-07
↳Metabolism of carbohydrates	181	16	3.34	4.79	+	5.40E-04
↳Metabolism	1245	61	22.96	2.66	+	2.97E-09
Translocation of GLUT4 to the plasma membrane	12	6	.22	27.12	+	1.82E-04
↳Membrane Trafficking	296	18	5.46	3.30	+	1.86E-02
↳Vesicle-mediated transport	319	23	5.88	3.91	+	6.08E-05
Glycolysis	15	7	.28	25.31	+	2.55E-05
Glycolysis	20	8	.37	21.69	+	7.91E-06
Formation of ATP by chemiosmotic coupling	17	5	.31	15.95	+	2.66E-02
TP53 Regulates Metabolic Genes	34	7	.63	11.17	+	5.82E-03
Lipid digestion, mobilization, and transport	61	11	1.12	9.78	+	3.96E-05
Platelet degranulation	102	18	1.88	9.57	+	2.10E-09
↳Response to elevated platelet cytosolic Ca2+	106	18	1.95	9.21	+	3.93E-09
↳Platelet activation, signaling and aggregation	200	22	3.69	5.97	+	6.23E-08
↳Hemostasis	330	30	6.08	4.93	+	1.87E-09
Degradation of the extracellular matrix	62	9	1.14	7.87	+	4.24E-03
↳Extracellular matrix organization	159	23	2.93	7.85	+	9.27E-11
Nonsense Mediated Decay (NMD) enhanced by the Exon Junction Complex (EJC)	80	9	1.48	6.10	+	3.16E-02
↳Nonsense-Mediated Decay (NMD)	80	9	1.48	6.10	+	3.16E-02
Unclassified	10480	119	193.24	.62	-	0.00E00

Supplementary Table S3

Appendix 2. Mascot search results of four changed proteins by tandem MS. Protein information of identification including sequence coverage, matched peptides and error are shown.

1. Apolipoprotein A1

MATRIX SCIENCE Mascot Search Results

Protein View

Match to: **IPI00580765** Score: **118**
Tax_Id=9031 Gene_Symbol=APOA1 Apolipoprotein A-I

Nominal mass (M_r): **30661**; Calculated pI value: **5.58**
NCBI BLAST search of IPI00580765 against nr
Unformatted sequence string for pasting into other applications

Fixed modifications: Carbamidomethyl (C)
Variable modifications: ICPL_0 (K),ICPL_0 (Protein N-term),ICPL_6 (K),ICPL_6 (Protein N-term),Oxidation (M)
Cleavage by Trypsin+GluC: cuts C-term side of ER unless next residue is P
Sequence Coverage: **51%**

Matched peptides shown in **Bold Red**

1 MRGVLVTLAV LFLTGTQAR**S FWQHDEPQTP LDRIRDMVDV YLETVKASGK**
51 **DAIAQFE**SSA VGKQLDLKLA DNLDTLSAAA AKLREDMAPY YKEVRE**MWLK**
101 **DTE**ALRAELT KDLEEVKE**KI RPFLDQFSAK WTEE**LEQYR**Q RLTPVAQELK**
151 **ELTKQKVELM QAKLTPVAEE** ARDRLRGHVE ELR**KNLAPYS DELRQKLSQK**
201 **LEE**IR**EKGIP QASEYQAKVM E**QLSNLR**EKM TPLVQE**FRER LTPYAENLKN
251 RLISFLDELQ KSVA

Show predicted peptides also

Sort Peptides By ◉ Residue Number ○ Increasing Mass ○ Decreasing Mass

Start - End	Observed	Mr(expt)	Mr(calc)	Delta	Miss	Sequence
20 - 33	878.3630	1754.7114	1754.8013	-0.0898	0	R.SFWQHDEPQTPLDR.I (Ions score 26)
20 - 33	878.4344	1754.8542	1754.8013	0.0530	0	R.SFWQHDEPQTPLDR.I (Ions score 29)
20 - 33	586.2846	1755.8320	1754.8013	1.0307	0	R.SFWQHDEPQTPLDR.I (Ions score 17)
34 - 43	626.8140	1251.6134	1251.6169	-0.0035	1	R.IRDMVDVYLE.T (Ions score 25)
34 - 43	626.8535	1251.6924	1251.6169	0.0755	1	R.IRDMVDVYLE.T (Ions score 19)
34 - 43	626.8631	1251.7116	1251.6169	0.0947	1	R.IRDMVDVYLE.T (Ions score 23)
44 - 57	558.8262	1673.4568	1673.8049	-0.3482	0	E.TVKASGKDAIAQFE.S 2 ICPL_0 (K) (Ions score 23)
44 - 57	837.9422	1673.8698	1673.8049	0.0649	0	E.TVKASGKDAIAQFE.S 2 ICPL_0 (K) (Ions score 26)
44 - 57	837.9459	1673.8772	1673.8049	0.0723	0	E.TVKASGKDAIAQFE.S 2 ICPL_0 (K) (Ions score 45)
44 - 57	843.9757	1685.9368	1685.8452	0.0917	0	E.TVKASGKDAIAQFE.S 2 ICPL_6 (K) (Ions score 23)
44 - 57	844.0061	1685.9976	1685.8452	0.1525	0	E.TVKASGKDAIAQFE.S 2 ICPL_6 (K) (Ions score 33)
44 - 57	844.0061	1685.9976	1685.8452	0.1525	0	E.TVKASGKDAIAQFE.S 2 ICPL_6 (K) (Ions score 59)
44 - 57	563.0185	1686.0337	1685.8452	0.1885	0	E.TVKASGKDAIAQFE.S 2 ICPL_6 (K) (Ions score 20)
44 - 57	563.2810	1686.8212	1685.8452	0.9760	0	E.TVKASGKDAIAQFE.S 2 ICPL_6 (K) (Ions score 14)
97 - 103	1033.3722	1032.3649	1032.4682	-0.1033	0	E.MWLKDTE.A ICPL_6 (K) (Ions score 10)
97 - 103	517.2096	1032.4046	1032.4682	-0.0635	0	E.MWLKDTE.A ICPL_6 (K) (Ions score 22)
119 - 134	735.7217	2204.1433	2204.0691	0.0742	1	E.KIRPFLDQFSAKWTEE.L 2 ICPL_0 (K) (Ions score 14)
140 - 148	521.6870	1041.3594	1040.5614	0.7980	1	R.QRLTPVAQE.L (Ions score 13)
140 - 148	521.7384	1041.4622	1040.5614	0.9008	1	R.QRLTPVAQE.L (Ions score 21)
142 - 151	616.8538	1231.6930	1231.6448	0.0482	1	R.LTPVAQELKE.L ICPL_0 (K) (Ions score 12)
142 - 151	616.8538	1231.6930	1231.6448	0.0482	1	R.LTPVAQELKE.L ICPL_0 (K) (Ions score 12)
142 - 151	619.8332	1237.6518	1237.6650	-0.0131	1	R.LTPVAQELKE.L ICPL_6 (K) (Ions score 14)
142 - 151	619.8332	1237.6518	1237.6650	-0.0131	1	R.LTPVAQELKE.L ICPL_6 (K) (Ions score 15)
152 - 158	528.1683	1054.3220	1054.5447	-0.2227	0	E.LTKQKVE.L 2 ICPL_0 (K) (Ions score 19)
152 - 158	528.1721	1054.3296	1054.5447	-0.2151	0	E.LTKQKVE.L 2 ICPL_0 (K) (Ions score 24)
152 - 158	534.1775	1066.3404	1066.5850	-0.2445	0	E.LTKQKVE.L 2 ICPL_6 (K) (Ions score 11)
159 - 170	717.9096	1433.8046	1433.7224	0.0822	1	E.LMQAKLTPVAEE.A ICPL_0 (K) (Ions score 29)
159 - 170	720.8424	1439.6702	1439.7426	-0.0723	1	E.LMQAKLTPVAEE.A ICPL_6 (K) (Ions score 29)
159 - 170	720.8846	1439.7546	1439.7426	0.0121	1	E.LMQAKLTPVAEE.A ICPL_6 (K) (Ions score 38)
184 - 192	571.2176	1140.4206	1140.5087	-0.0881	0	R.KNLAPYSDE.L ICPL_0 (K) (Ions score 17)
184 - 192	571.2854	1140.5562	1140.5087	0.0475	0	R.KNLAPYSDE.L ICPL_0 (K) (Ions score 7)
184 - 194	470.8346	1409.4820	1409.6939	-0.2119	1	R.KNLAPYSDELR.Q ICPL_0 (K) (Ions score 25)
184 - 194	705.8726	1409.7306	1409.6939	0.0368	1	R.KNLAPYSDELR.Q ICPL_0 (K) (Ions score 37)
184 - 194	705.8726	1409.7306	1409.6939	0.0368	1	R.KNLAPYSDELR.Q ICPL_0 (K) (Ions score 36)
184 - 194	705.8726	1409.7306	1409.6939	0.0368	1	R.KNLAPYSDELR.Q ICPL_0 (K) (Ions score 42)
184 - 194	705.8726	1409.7306	1409.6939	0.0368	1	R.KNLAPYSDELR.Q ICPL_0 (K) (Ions score 46)
184 - 194	708.8614	1415.7082	1415.7140	-0.0058	1	R.KNLAPYSDELR.Q ICPL_6 (K) (Ions score 13)
195 - 203	604.7484	1207.4822	1206.6244	0.8578	1	R.QKLSQKLEE.I ICPL_0 (K) (Ions score 11)
195 - 203	662.8150	1323.6154	1323.6861	-0.0707	1	R.QKLSQKLEE.I 2 ICPL_6 (K) (Ions score 17)
195 - 203	662.8565	1323.6984	1323.6861	0.0123	1	R.QKLSQKLEE.I 2 ICPL_6 (K) (Ions score 6)
206 - 214	535.2317	1068.4488	1068.5183	-0.0694	1	R.EKGIPQASE.Y ICPL_6 (K) (Ions score 18)
215 - 221	487.1980	972.3814	972.4375	-0.0560	0	E.YQAKVME.Q ICPL_0 (K) (Ions score 21)

215 - 221	490.1566	978.2986	978.4576	-0.1590	0	**E.YQAKVME.Q**	ICPL_6 (K) (Ions score 25)
215 - 221	490.1566	978.2986	978.4576	-0.1590	0	**E.YQAKVME.Q**	ICPL_6 (K) (Ions score 31)
215 - 221	979.3578	978.3505	978.4576	-0.1071	0	**E.YQAKVME.Q**	ICPL_6 (K) (Ions score 15)
228 - 236	593.3112	1184.6078	1184.5843	0.0236	1	**R.EKMTPLVQE.F**	ICPL_6 (K) (Ions score 16)

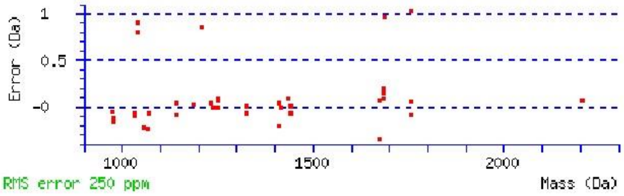

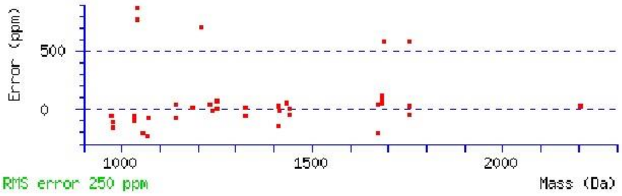

Mascot: http://www.matrixscience.com/

2. Cystatin

Mascot Search Results

Protein View

Match to: **IPI00576782** Score: **211**
Tax_Id=9031 Gene_Symbol=CST3 Cystatin

Nominal mass (M_r): **15562;** Calculated pI value: **7.60**
NCBI BLAST search of IPI00576782 against nr
Unformatted sequence string for pasting into other applications

Fixed modifications: Carbamidomethyl (C)
Variable modifications: ICPL_0 (K),ICPL_0 (Protein N-term),ICPL_6 (K),ICPL_6 (Protein N-term),Oxidation (M)
Cleavage by Trypsin+GluC: cuts C-term side of ER unless next residue is P
Sequence Coverage: **41%**

Matched peptides shown in **Bold Red**

```
  1 MAGARGCVVL LAAALMLVGA VLGSEDRSRL LGAPVPVDEN DEGLQRALQF
 51 AMAEYNRASN DKYSSRVVRV ISAKRQLVSG IKYILQVEIG RTTCPKSSGD
101 LQSCEFHDEP EMAKYTTCTF VVYSIPWLNQ IKLLESKCQ
```

Show predicted peptides also

Sort Peptides By ◉ Residue Number ○ Increasing Mass ○ Decreasing Mass

Start - End	Observed	Mr(expt)	Mr(calc)	Delta	Miss	Sequence
30 - 39	1009.4981	1008.4908	1008.5492	-0.0583	0	R.LLGAPVPVDE.N (Ions score 20)
30 - 39	1009.5210	1008.5137	1008.5492	-0.0354	0	R.LLGAPVPVDE.N (Ions score 23)
30 - 42	684.3103	1366.6060	1366.6616	-0.0556	1	R.LLGAPVPVDENDE.G (Ions score 43)
30 - 42	684.3447	1366.6748	1366.6616	0.0132	1	R.LLGAPVPVDENDE.G (Ions score 54)
30 - 42	684.3516	1366.6886	1366.6616	0.0270	1	R.LLGAPVPVDENDE.G (Ions score 17)
47 - 54	880.4014	879.3941	879.4160	-0.0219	0	R.ALQFAMAE.Y (Ions score 14)
47 - 57	657.2937	1312.5728	1312.6234	-0.0505	1	R.ALQFAMAEYNR.A (Ions score 31)
47 - 57	657.3157	1312.6168	1312.6234	-0.0065	1	R.ALQFAMAEYNR.A (Ions score 52)
47 - 57	657.3157	1312.6168	1312.6234	-0.0065	1	R.ALQFAMAEYNR.A (Ions score 57)
76 - 88	797.9250	1593.8354	1593.8766	-0.0412	0	R.QLVSGIKYILQVE.I ICPL_0 (K) (Ions score 28)
76 - 88	797.9502	1593.8858	1593.8766	0.0092	0	R.QLVSGIKYILQVE.I ICPL_0 (K) (Ions score 6)
76 - 88	797.9725	1593.9304	1593.8766	0.0538	0	R.QLVSGIKYILQVE.I ICPL_0 (K) (Ions score 38)
76 - 88	798.0048	1593.9950	1593.8766	0.1184	0	R.QLVSGIKYILQVE.I ICPL_0 (K) (Ions score 53)
76 - 88	532.5421	1594.6045	1593.8766	0.7278	0	R.QLVSGIKYILQVE.I ICPL_0 (K) (Ions score 34)
76 - 88	532.5421	1594.6045	1593.8766	0.7278	0	R.QLVSGIKYILQVE.I ICPL_0 (K) (Ions score 32)
92 - 105	837.8532	1673.6918	1673.6662	0.0257	0	R.TTCPKSSGDLQSCE.F ICPL_0 (K) (Ions score 49)
92 - 105	837.8622	1673.7098	1673.6662	0.0437	0	R.TTCPKSSGDLQSCE.F ICPL_0 (K) (Ions score 57)
92 - 105	837.8964	1673.7782	1673.6662	0.1121	0	R.TTCPKSSGDLQSCE.F ICPL_0 (K) (Ions score 47)
92 - 105	840.8700	1679.7254	1679.6863	0.0392	0	R.TTCPKSSGDLQSCE.F ICPL_6 (K) (Ions score 30)
92 - 111	1214.9326	2427.8506	2427.9584	-0.1077	1	R.TTCPKSSGDLQSCEFHDEPE.M ICPL_0 (K) (Ions score 48)
92 - 111	1215.0903	2428.1660	2427.9584	0.2077	1	R.TTCPKSSGDLQSCEFHDEPE.M ICPL_0 (K) (Ions score 30)
92 - 111	1215.0903	2428.1660	2427.9584	0.2077	1	R.TTCPKSSGDLQSCEFHDEPE.M ICPL_0 (K) (Ions score 30)
92 - 111	1215.6187	2429.2228	2427.9584	1.2645	1	R.TTCPKSSGDLQSCEFHDEPE.M ICPL_0 (K) (Ions score 36)

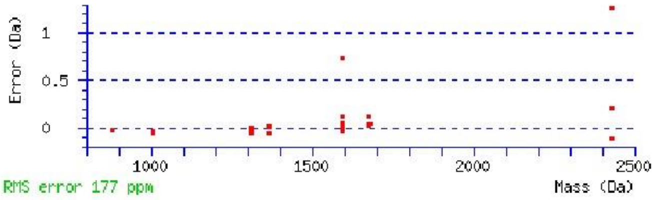

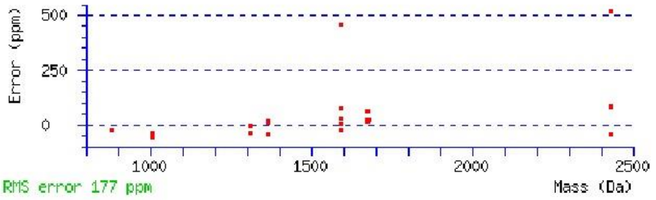

Mascot: http://www.matrixscience.com/

3. Ovotransferrin

{MATRIX SCIENCE} Mascot Search Results

Protein View

Match to: **IPI00971372** Score: **597**
Tax_Id=9031 Gene_Symbol=TF Ovotransferrin BC type

Nominal mass (M_r): **79588**; Calculated pI value: **7.08**
NCBI BLAST search of IPI00971372 against nr
Unformatted sequence string for pasting into other applications

Fixed modifications: Carbamidomethyl (C)
Variable modifications: ICPL_0 (K),ICPL_0 (Protein N-term),ICPL_6 (K),ICPL_6 (Protein N-term),Oxidation (M)
Cleavage by Trypsin+GluC: cuts C-term side of ER unless next residue is P
Sequence Coverage: **47%**

Matched peptides shown in **Bold Red**

1 MKLILCTVLS LGIAAVCFAA PPKSVIRWCT ISSPEEKKCN NLRDLTQQER
51 IALTCVQKAT YLDCIKAIAN NE**ADAISLDG GQVFEAGLAP YKLKPXAAEV**
101 **YE**HTEGSTTS YYAVAVVKKG TE**FTVNDLQG KTSCHTGLGR SAGWNIPIGT**
151 **LIHR**GAIEWE GIESGSVE**QA VAKFFSASCV PGATIE**QKLC R**QCKGDPKTK**
201 **CAR**NAPYSGY SGAFHCLKDG KGDVAFVKHT TVNENAPDQK DEYE**LLCLDG**
251 **SRQPVDNYKT CNWAR**VAAHA VVARDDNKVE DIWSFLSKAQ SDFGVDTKSD
301 FHLFGPPGKK DPVLKDLLFK DSAIMLKR**VP SLMDSQLYLG FEYYSAIQSM**
351 **RKDQLTPSPR** ENR**IQWCAVG KDE**KSKCDR**W SVVSNGDVEC TVVDETKDCI**
401 **IKIMKGEADA VALDGGLVYT AGVCGLVPVM AE**RYDDE**SQC SKTDERPASY**
451 **FAVAVARKDS NVNWNNLKGK KSCHTAVGR**T AGWVIPMGLI HNR**TGTCNFB**
501 **EYFSE**GCAPG SPPNSR**LCQL CQGSGGIPPE KCVASSHEKY FGYTGALRCL**
551 **VE**KGDVAFIQ HSTVEE**NTGG KNKADWAKNL QMDDFE**LLCT DGRRANVMDY
601 RECNLAE**VPT HAVVVRPE**KA NKIRDLLERQ EKRFGVNGSE **KSKFMMFE**SQ
651 NKDLLFKDLT KCLFKVREGT TYKEFLGDKF YTVISNLKTC NPSDILQMCS
701 FLEGK

Show predicted peptides also

Sort Peptides By ◉ Residue Number ○ Increasing Mass ○ Decreasing Mass

Start - End	Observed	Mr(expt)	Mr(calc)	Delta	Miss	Sequence
73 - 85	661.3840	1320.7534	1320.6198	0.1337	0	E.ADAISLDGGQVFE.A (Ions score 68)
73 - 85	661.3840	1320.7534	1320.6198	0.1337	0	E.ADAISLDGGQVFE.A (Ions score 57)
86 - 99	758.3547	1514.6948	1513.8293	0.8656	0	E.AGLAPYKLKPWAAE.V (Ions score 6)
86 - 99	826.4892	1650.9638	1650.8769	0.0869	0	E.AGLAPYKLKPIAAE.V 2 ICPL_0 (K) (Ions score 20)
86 - 99	826.4892	1650.9638	1650.8769	0.0869	0	E.AGLAPYKLKPIAAE.V 2 ICPL_0 (K) (Ions score 24)
86 - 99	832.4687	1662.9228	1662.9172	0.0057	0	E.AGLAPYKLKPIAAE.V 2 ICPL_6 (K) (Ions score 20)
86 - 99	832.4804	1662.9462	1662.9172	0.0291	0	E.AGLAPYKLKPIAAE.V 2 ICPL_6 (K) (Ions score 9)
86 - 99	832.4930	1662.9714	1662.9172	0.0543	0	E.AGLAPYKLKPIAAE.V 2 ICPL_6 (K) (Ions score 15)
86 - 102	972.3392	1942.6638	1943.0499	-0.3861	1	E.AGLAPYKLKPIAAEVYE.H ICPL_6 (K) (Ions score 20)
86 - 102	1022.0112	2042.0078	2042.0513	-0.0434	1	E.AGLAPYKLKPIAAEVYE.H 2 ICPL_0 (K) (Ions score 36)
86 - 102	1022.0112	2042.0078	2042.0513	-0.0434	1	E.AGLAPYKLKPIAAEVYE.H 2 ICPL_0 (K) (Ions score 23)
86 - 102	1022.0167	2042.0188	2042.0513	-0.0324	1	E.AGLAPYKLKPIAAEVYE.H 2 ICPL_0 (K) (Ions score 43)
86 - 102	681.6990	2042.0752	2042.0513	0.0239	1	E.AGLAPYKLKPIAAEVYE.H 2 ICPL_0 (K) (Ions score 18)
86 - 102	1027.9861	2053.9576	2054.0915	-0.1339	1	E.AGLAPYKLKPIAAEVYE.H 2 ICPL_6 (K) (Ions score 39)
86 - 102	685.6679	2053.9819	2054.0915	-0.1097	1	E.AGLAPYKLKPIAAEVYE.H 2 ICPL_6 (K) (Ions score 20)
86 - 102	685.8172	2054.4298	2054.0915	0.3383	1	E.AGLAPYKLKPIAAEVYE.H 2 ICPL_6 (K) (Ions score 18)
123 - 140	699.2996	2094.8770	2094.9906	-0.1136	0	E.FTVNDLQGKTSCHTGLGR.S ICPL_0 (K) (Ions score 28)
123 - 140	1048.4458	2094.8770	2094.9906	-0.1135	0	E.FTVNDLQGKTSCHTGLGR.S ICPL_0 (K) (Ions score 20)
123 - 140	699.3319	2094.9739	2094.9906	-0.0167	0	E.FTVNDLQGKTSCHTGLGR.S ICPL_0 (K) (Ions score 17)
123 - 140	1048.5651	2095.1156	2094.9906	0.1251	0	E.FTVNDLQGKTSCHTGLGR.S ICPL_0 (K) (Ions score 39)
141 - 154	767.7754	1533.5362	1533.8416	-0.3053	0	R.SAGWNIPIGTLIHR.G (Ions score 10)
141 - 154	512.2477	1533.7213	1533.8416	-0.1203	0	R.SAGWNIPIGTLIHR.G (Ions score 36)
141 - 154	512.2501	1533.7285	1533.8416	-0.1131	0	R.SAGWNIPIGTLIHR.G (Ions score 42)
141 - 154	767.8876	1533.7606	1533.8416	-0.0809	0	R.SAGWNIPIGTLIHR.G (Ions score 41)
141 - 154	512.2656	1533.7750	1533.8416	-0.0666	0	R.SAGWNIPIGTLIHR.G (Ions score 47)
141 - 154	512.2704	1533.7894	1533.8416	-0.0522	0	R.SAGWNIPIGTLIHR.G (Ions score 45)
141 - 154	767.9356	1533.8566	1533.8416	0.0151	0	R.SAGWNIPIGTLIHR.G (Ions score 26)
141 - 154	767.9363	1533.8580	1533.8416	0.0165	0	R.SAGWNIPIGTLIHR.G (Ions score 20)
141 - 154	767.9382	1533.8618	1533.8416	0.0203	0	R.SAGWNIPIGTLIHR.G (Ions score 18)
141 - 154	767.9824	1533.9502	1533.8416	0.1087	0	R.SAGWNIPIGTLIHR.G (Ions score 22)
141 - 154	512.3241	1533.9505	1533.8416	0.1089	0	R.SAGWNIPIGTLIHR.G (Ions score 36)
141 - 154	768.0103	1534.0060	1533.8416	0.1645	0	R.SAGWNIPIGTLIHR.G (Ions score 17)
169 - 186	994.4669	1986.9192	1986.9510	-0.0317	0	E.QAVAKFFSASCVPGATIE.Q ICPL_0 (K) (Ions score 19)
169 - 186	994.4710	1986.9274	1986.9510	-0.0235	0	E.QAVAKFFSASCVPGATIE.Q ICPL_0 (K) (Ions score 26)
169 - 186	994.5040	1986.9934	1986.9510	0.0425	0	E.QAVAKFFSASCVPGATIE.Q ICPL_0 (K) (Ions score 7)
169 - 186	994.9605	1987.9064	1986.9510	0.9555	0	E.QAVAKFFSASCVPGATIE.Q ICPL_0 (K) (Ions score 22)
169 - 186	997.4596	1992.9046	1992.9711	-0.0664	0	E.QAVAKFFSASCVPGATIE.Q ICPL_6 (K) (Ions score 33)
169 - 186	997.4596	1992.9046	1992.9711	-0.0664	0	E.QAVAKFFSASCVPGATIE.Q ICPL_6 (K) (Ions score 31)
192 - 203	882.3409	1762.6672	1762.7668	-0.0996	0	R.QCKGDPKTKCAR.N 3 ICPL_0 (K) (Ions score 31)
192 - 203	882.3843	1762.7540	1762.7668	-0.0128	0	R.QCKGDPKTKCAR.N 3 ICPL_0 (K) (Ions score 10)
192 - 203	891.4439	1780.8732	1780.8272	0.0461	0	R.QCKGDPKTKCAR.N 3 ICPL_6 (K) (Ions score 18)
192 - 203	891.4439	1780.8732	1780.8272	0.0461	0	R.QCKGDPKTKCAR.N 3 ICPL_6 (K) (Ions score 19)
192 - 203	594.6317	1780.8733	1780.8272	0.0461	0	R.QCKGDPKTKCAR.N 3 ICPL_6 (K) (Ions score 11)
245 - 252	467.1296	932.2446	932.4749	-0.2303	0	E.LLCLDGSR.Q (Ions score 41)
245 - 252	467.1296	932.2446	932.4749	-0.2303	0	E.LLCLDGSR.Q (Ions score 41)
245 - 252	467.1613	932.3080	932.4749	-0.1669	0	E.LLCLDGSR.Q (Ions score 32)

253 - 265	878.8745	1755.7344	1755.7787	-0.0443	0	R.QPVDNYKTCNWAR.V	ICPL_0 (K) (Ions score 19)
253 - 265	586.2521	1755.7345	1755.7787	-0.0443	0	R.QPVDNYKTCNWAR.V	ICPL_0 (K) (Ions score 16)
253 - 265	586.5616	1756.6630	1755.7787	0.8842	0	R.QPVDNYKTCNWAR.V	ICPL_0 (K) (Ions score 18)
253 - 265	586.5952	1756.7638	1755.7787	0.9850	0	R.QPVDNYKTCNWAR.V	ICPL_0 (K) (Ions score 24)
253 - 265	586.5952	1756.7638	1755.7787	0.9850	0	R.QPVDNYKTCNWAR.V	ICPL_0 (K) (Ions score 23)
253 - 265	881.9121	1761.8096	1761.7989	0.0108	0	R.QPVDNYKTCNWAR.V	ICPL_6 (K) (Ions score 51)
253 - 265	881.9121	1761.8096	1761.7989	0.0108	0	R.QPVDNYKTCNWAR.V	ICPL_6 (K) (Ions score 52)
329 - 342	799.9117	1597.8088	1597.7698	0.0391	0	R.VPSLMDSQLYLGFE.Y	(Ions score 46)
329 - 342	799.9222	1597.8298	1597.7698	0.0601	0	R.VPSLMDSQLYLGFE.Y	(Ions score 31)
329 - 342	799.9239	1597.8332	1597.7698	0.0635	0	R.VPSLMDSQLYLGFE.Y	(Ions score 47)
343 - 351	559.6952	1117.3758	1117.5226	-0.1467	0	E.YYSAIQSMR.K	(Ions score 46)
343 - 351	559.7082	1117.4018	1117.5226	-0.1208	0	E.YYSAIQSMR.K	(Ions score 49)
352 - 360	573.7278	1145.4410	1145.5829	-0.1419	0	R.KDQLTPSPR.E	ICPL_0 (K) (Ions score 49)
352 - 360	573.7511	1145.4876	1145.5829	-0.0953	0	R.KDQLTPSPR.E	ICPL_0 (K) (Ions score 40)
352 - 360	573.7511	1145.4876	1145.5829	-0.0953	0	R.KDQLTPSPR.E	ICPL_0 (K) (Ions score 49)
352 - 360	573.7637	1145.5128	1145.5829	-0.0701	0	R.KDQLTPSPR.E	ICPL_0 (K) (Ions score 44)
352 - 360	573.7697	1145.5248	1145.5829	-0.0581	0	R.KDQLTPSPR.E	ICPL_0 (K) (Ions score 46)
352 - 360	576.7552	1151.4958	1151.6030	-0.1072	0	R.KDQLTPSPR.E	ICPL_6 (K) (Ions score 56)
352 - 360	576.7864	1151.5582	1151.6030	-0.0448	0	R.KDQLTPSPR.E	ICPL_6 (K) (Ions score 50)
364 - 373	658.8079	1315.6012	1315.5963	0.0050	0	R.IQWCAVGKDE.K	ICPL_6 (K) (Ions score 26)
364 - 373	658.8079	1315.6012	1315.5963	0.0050	0	R.IQWCAVGKDE.K	ICPL_6 (K) (Ions score 18)
364 - 373	658.8334	1315.6522	1315.5963	0.0560	0	R.IQWCAVGKDE.K	ICPL_6 (K) (Ions score 16)
380 - 395	897.8936	1793.7726	1793.7778	-0.0052	1	R.WSVVSNGDVECTVVDE.T	(Ions score 16)
380 - 395	898.4433	1794.8720	1793.7778	1.0942	1	R.WSVVSNGDVECTVVDE.T	(Ions score 26)
390 - 407	1178.0666	2354.1186	2354.1052	0.0134	1	E.CTVVDETKDCIIKIMKGE.A	ICPL_0 (K); ICPL_6 (K) (Ions score 9)
396 - 407	584.2266	1749.6580	1749.8218	-0.1639	0	E.TKDCIIKIMKGE.A	3 ICPL_0 (K) (Ions score 29)
396 - 407	584.3337	1749.9793	1749.8218	0.1574	0	E.TKDCIIKIMKGE.A	3 ICPL_0 (K) (Ions score 41)
396 - 432	1368.3469	4102.0189	4102.0326	-0.0138	1	E.TKDCIIKIMKGEADAVALDGGLVYTAGVCGLVPVMAE.R	2 ICPL_6 (K); Oxidation (M) (Ions score 11)
438 - 457	783.7053	2348.0941	2347.1015	0.9926	1	E.SQCSKTDERPASYFAVAVAR.K	ICPL_0 (K) (Ions score 24)
438 - 457	783.7053	2348.0941	2347.1015	0.9926	1	E.SQCSKTDERPASYFAVAVAR.K	ICPL_0 (K) (Ions score 29)
458 - 479	986.4173	2956.2301	2956.4381	-0.2080	0	R.KDSNVNWNNLKGKKSCHTAVGR.T	4 ICPL_6 (K) (Ions score 66)
458 - 479	986.5812	2956.7218	2956.4381	0.2837	0	R.KDSNVNWNNLKGKKSCHTAVGR.T	4 ICPL_6 (K) (Ions score 33)
494 - 505	735.2555	1468.4964	1468.5453	-0.0488	1	R.TGTCNFDEYFSE.G	(Ions score 18)
494 - 505	735.2815	1468.5484	1468.5453	0.0032	1	R.TGTCNFDEYFSE.G	(Ions score 33)
494 - 505	735.3012	1468.5878	1468.5453	0.0426	1	R.TGTCNFDEYFSE.G	(Ions score 30)
517 - 538	840.3527	2518.0363	2518.1039	-0.0677	1	R.LCQLCQGSGGIPPEKCVASSHE.K	ICPL_0 (K) (Ions score 30)
517 - 538	842.3937	2524.1593	2524.1241	0.0352	1	R.LCQLCQGSGGIPPEKCVASSHE.K	ICPL_6 (K) (Ions score 13)
517 - 538	842.3937	2524.1593	2524.1241	0.0352	1	R.LCQLCQGSGGIPPEKCVASSHE.K	ICPL_6 (K) (Ions score 28)
539 - 548	640.8276	1279.6406	1279.6349	0.0057	0	E.KYFGYTGALR.C	ICPL_0 (K) (Ions score 43)
539 - 548	640.8290	1279.6434	1279.6349	0.0085	0	E.KYFGYTGALR.C	ICPL_0 (K) (Ions score 12)
539 - 548	640.8581	1279.7016	1279.6349	0.0667	0	E.KYFGYTGALR.C	ICPL_0 (K) (Ions score 43)
539 - 548	640.8581	1279.7016	1279.6349	0.0667	0	E.KYFGYTGALR.C	ICPL_0 (K) (Ions score 46)
539 - 548	640.9522	1279.8898	1279.6349	0.2549	0	E.KYFGYTGALR.C	ICPL_0 (K) (Ions score 13)
539 - 548	643.8466	1285.6786	1285.6551	0.0236	0	E.KYFGYTGALR.C	ICPL_6 (K) (Ions score 8)
539 - 552	559.9263	1676.7571	1675.8392	0.9179	1	E.KYFGYTGALRCLVE.K	(Ions score 12)
567 - 586	866.7033	2597.0881	2596.1077	0.9803	0	E.NTGGKNKADWAKNLQMDDFE.L	3 ICPL_0 (K) (Ions score 15)
567 - 586	866.7033	2597.0881	2596.1077	0.9803	0	E.NTGGKNKADWAKNLQMDDFE.L	3 ICPL_0 (K) (Ions score 54)
567 - 586	872.3646	2614.0720	2614.1681	-0.0961	0	E.NTGGKNKADWAKNLQMDDFE.L	3 ICPL_6 (K) (Ions score 34)
567 - 586	872.3646	2614.0720	2614.1681	-0.0961	0	E.NTGGKNKADWAKNLQMDDFE.L	3 ICPL_6 (K) (Ions score 10)
608 - 618	602.3203	1202.6260	1202.6772	-0.0511	0	E.VPTHAVVVRPE.K	(Ions score 54)
641 - 648	629.2952	1256.5758	1256.5358	0.0400	0	E.KSKFMMFE.S	2 ICPL_0 (K) (Ions score 31)
641 - 648	629.2952	1256.5758	1256.5358	0.0400	0	E.KSKFMMFE.S	2 ICPL_0 (K) (Ions score 25)
641 - 648	635.2760	1268.5374	1268.5761	-0.0386	0	E.KSKFMMFE.S	2 ICPL_6 (K) (Ions score 18)

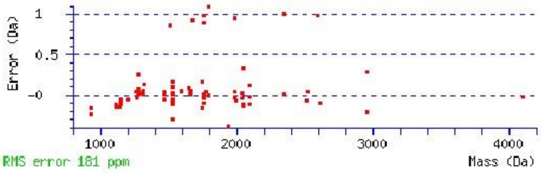

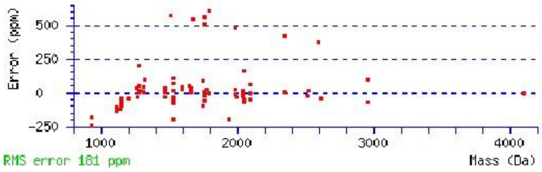

Mascot: http://www.matrixscience.com/

4. Purpurin

Mascot Search Results

Protein View

Match to: **IPI00600469** Score: **596**
Tax_Id=9031 Gene_Symbol=LOC396454 Purpurin

Nominal mass (M_r): **22250**; Calculated pI value: **4.77**
NCBI BLAST search of IPI00600469 against nr
Unformatted sequence string for pasting into other applications

Fixed modifications: Carbamidomethyl (C)
Variable modifications: ICPL_0 (K),ICPL_0 (Protein N-term),ICPL_6 (K),ICPL_6 (Protein N-term),Oxidation (M)
Cleavage by Trypsin+GluC: cuts C-term side of ER unless next residue is P
Sequence Coverage: **34%**

Matched peptides shown in **Bold Red**

```
  1 MKYAQYVFLA SIFSAVEYSL AQTCAVDSFS VKDNFDPKRY AGKWYALAKK
 51 DPEGLFLQDN ISAEYTVEED GTMTASSKGR VKLFGFWVIC ADMAAQYTVP
101 DPTTPAKMYM TYQGLASYLS SGGDNYWVID TDYDNYAITY ACRSLKEDGS
151 CDDGYSLIFS RNPRGLPPAI QRIVRQKQEE ICMSGQFQPV LQSGAC
```

Show predicted peptides also

Sort Peptides By ◉ Residue Number ○ Increasing Mass ○ Decreasing Mass

Start - End	Observed	Mr(expt)	Mr(calc)	Delta	Miss	Sequence
40 - 53	977.9780	1953.9414	1953.9050	0.0365	0	R.YAGKWYALAKKDPE.G 3 ICPL_0 (K) (Ions score 38)
40 - 53	977.9780	1953.9414	1953.9050	0.0365	0	R.YAGKWYALAKKDPE.G 3 ICPL_0 (K) (Ions score 41)
40 - 53	652.3443	1954.0111	1953.9050	0.1061	0	R.YAGKWYALAKKDPE.G 3 ICPL_0 (K) (Ions score 12)
40 - 53	652.3443	1954.0111	1953.9050	0.1061	0	R.YAGKWYALAKKDPE.G 3 ICPL_0 (K) (Ions score 15)
40 - 53	652.3447	1954.0123	1953.9050	0.1073	0	R.YAGKWYALAKKDPE.G 3 ICPL_0 (K) (Ions score 11)
40 - 53	986.9757	1971.9368	1971.9653	-0.0285	0	R.YAGKWYALAKKDPE.G 3 ICPL_6 (K) (Ions score 38)
40 - 53	986.9757	1971.9368	1971.9653	-0.0285	0	R.YAGKWYALAKKDPE.G 3 ICPL_6 (K) (Ions score 48)
40 - 53	658.3288	1971.9646	1971.9653	-0.0008	0	R.YAGKWYALAKKDPE.G 3 ICPL_6 (K) (Ions score 13)
40 - 53	658.3288	1971.9646	1971.9653	-0.0008	0	R.YAGKWYALAKKDPE.G 3 ICPL_6 (K) (Ions score 14)
54 - 64	603.7847	1205.5548	1205.5928	-0.0380	0	E.GLFLQDNISAE.Y (Ions score 66)
54 - 64	603.7847	1205.5548	1205.5928	-0.0380	0	E.GLFLQDNISAE.Y (Ions score 54)
54 - 64	603.7939	1205.5732	1205.5928	-0.0196	0	E.GLFLQDNISAE.Y (Ions score 9)
144 - 161	683.6235	2047.8487	2047.9157	-0.0670	1	R.SLKEDGSCDDGYSLIFSR.N (Ions score 17)
144 - 161	1024.9317	2047.8488	2047.9157	-0.0668	1	R.SLKEDGSCDDGYSLIFSR.N (Ions score 69)
144 - 161	1024.9459	2047.8772	2047.9157	-0.0384	1	R.SLKEDGSCDDGYSLIFSR.N (Ions score 79)
144 - 161	1024.9825	2047.9504	2047.9157	0.0348	1	R.SLKEDGSCDDGYSLIFSR.N (Ions score 94)
144 - 161	718.6648	2152.9726	2152.9371	0.0354	1	R.SLKEDGSCDDGYSLIFSR.N ICPL_0 (K) (Ions score 24)
144 - 161	718.6691	2152.9855	2152.9371	0.0483	1	R.SLKEDGSCDDGYSLIFSR.N ICPL_0 (K) (Ions score 38)
144 - 161	718.6704	2152.9894	2152.9371	0.0522	1	R.SLKEDGSCDDGYSLIFSR.N ICPL_0 (K) (Ions score 41)
144 - 161	718.6704	2152.9894	2152.9371	0.0522	1	R.SLKEDGSCDDGYSLIFSR.N ICPL_0 (K) (Ions score 29)
144 - 161	1077.5355	2153.0564	2152.9371	0.1193	1	R.SLKEDGSCDDGYSLIFSR.N ICPL_0 (K) (Ions score 105)
144 - 161	1077.5355	2153.0564	2152.9371	0.1193	1	R.SLKEDGSCDDGYSLIFSR.N ICPL_0 (K) (Ions score 92)
144 - 161	720.7387	2159.1943	2158.9573	0.2370	1	R.SLKEDGSCDDGYSLIFSR.N ICPL_6 (K) (Ions score 10)
148 - 161	796.3584	1590.7022	1590.6620	0.0402	0	E.DGSCDDGYSLIFSR.N (Ions score 71)
148 - 161	796.3584	1590.7022	1590.6620	0.0402	0	E.DGSCDDGYSLIFSR.N (Ions score 86)
148 - 161	796.3585	1590.7024	1590.6620	0.0404	0	E.DGSCDDGYSLIFSR.N (Ions score 74)
165 - 172	426.1552	850.2958	850.5025	-0.2066	0	R.GLPPAIQR.I (Ions score 29)
165 - 172	426.1568	850.2990	850.5025	-0.2034	0	R.GLPPAIQR.I (Ions score 28)
165 - 172	426.1568	850.2990	850.5025	-0.2034	0	R.GLPPAIQR.I (Ions score 25)
181 - 196	891.9078	1781.8010	1781.7899	0.0111	0	E.ICMSGQFQPVLQSGAC.- (Ions score 52)
181 - 196	891.9158	1781.8170	1781.7899	0.0271	0	E.ICMSGQFQPVLQSGAC.- (Ions score 43)
181 - 196	891.9460	1781.8774	1781.7899	0.0875	0	E.ICMSGQFQPVLQSGAC.- (Ions score 25)
181 - 196	891.9566	1781.8986	1781.7899	0.1087	0	E.ICMSGQFQPVLQSGAC.- (Ions score 52)
181 - 196	891.9623	1781.9100	1781.7899	0.1201	0	E.ICMSGQFQPVLQSGAC.- (Ions score 25)
181 - 196	891.9653	1781.9160	1781.7899	0.1261	0	E.ICMSGQFQPVLQSGAC.- (Ions score 32)
181 - 196	891.9783	1781.9420	1781.7899	0.1521	0	E.ICMSGQFQPVLQSGAC.- (Ions score 19)
181 - 196	891.9783	1781.9420	1781.7899	0.1521	0	E.ICMSGQFQPVLQSGAC.- (Ions score 12)

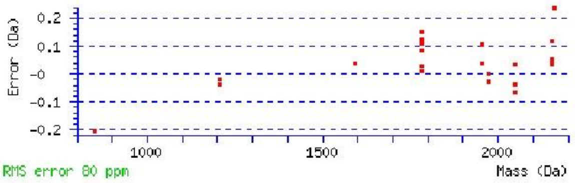

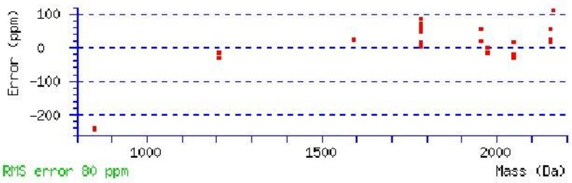

Supplement: Supplementary file 1 — supplementary information [file 41598_2017_12650_MOESM1_ESM.pdf]
